# Adaptive Evolution of Feline Coronavirus Genes Based on Selection Analysis

**DOI:** 10.1155/2020/9089768

**Published:** 2020-08-13

**Authors:** Hongyue Xia, Xibao Li, Wenliang Zhao, Shuran Jia, Xiaoqing Zhang, David M. Irwin, Shuyi Zhang

**Affiliations:** ^1^College of Animal Science and Veterinary Medicine, Shenyang Agricultural University, Shenyang 110866, China; ^2^Department of Laboratory Medicine and Pathobiology, University of Toronto, Toronto, Ontario, Canada M5S 1A8

## Abstract

**Purpose:**

We investigated sequences of the feline coronaviruses (FCoV), which include feline enteric coronavirus (FECV) and feline infectious peritonitis virus (FIPV), from China and other countries to gain insight into the adaptive evolution of this virus.

**Methods:**

Ascites samples from 31 cats with suspected FIP and feces samples from 8 healthy cats were screened for the presence of FCoV. Partial viral genome sequences, including parts of the nsp12-nsp14, S, N, and 7b genes, were obtained and aligned with additional sequences obtained from the GenBank database. Bayesian phylogenetic analysis was conducted, and the possibility of recombination within these sequences was assessed. Analysis of the levels of selection pressure experienced by these sequences was assessed using methods on both the PAML and Datamonkey platforms.

**Results:**

Of the 31 cats investigated, two suspected FIP cats and one healthy cat tested positive for FCoV. Phylogenetic analysis showed that all of the sequences from mainland China cluster together with a few sequences from the Netherlands as a distinct clade when analyzed with FCoV sequences from other countries. Fewer than 3 recombination breakpoints were detected in the nsp12-nsp14, S, N, and 7b genes, suggesting that analyses for positive selection could be conducted. A total of 4, 12, 4, and 4 positively selected sites were detected in the nsp12-nsp14, S, N, and 7b genes, respectively, with the previously described site 245 of the S gene, which distinguishes FIPV from FECV, being a positive selection site. Conversely, 106, 168, 25, and 17 negative selection sites in the nsp12-14, S, N, and 7b genes, respectively, were identified.

**Conclusion:**

Our study provides evidence that the FCoV genes encoding replicative, entry, and virulence proteins potentially experienced adaptive evolution. A greater number of sites in each gene experienced negative rather than positive selection, which suggests that most of the protein sequence must be conservatively maintained for virus survival. A few of the sites showing evidence of positive selection might be associated with the more severe pathology of FIPV or help these viruses survive other harmful conditions.

## 1. Introduction

Feline coronaviruses (FCoV) belong to the genus Alpha-coronavirus within the subfamily Coronavirinae of the family Coronaviridae in the order Nidovirales [1]. FCoV include the feline enteric coronavirus (FECV) and the feline infectious peritonitis virus (FIPV) [2]. Similar to other coronaviruses, such as the SARS and MERS viruses, FIPV infections are distributed worldwide and can cause a fatal pathogenic disease FIP in their hosts, thus, seriously endangering the life and health of cats [3]. However, the more common variant, FECV, causes an asymptomatic or mild enteric infection [3]. FIPV and FECV are antigenically divided into two types (I and II) based on a difference in the nucleotide sequence of the S gene, which encodes the spike protein [4]. Most natural cases of feline coronavirus infection are type I FCoV; however, these viruses poorly propagate in cell culture, whereas type II FCoV viruses can grow in several different cell lines [5].

The pathogenesis of FIP is not yet completely understood. It has been suggested that large viral quasispecies of FCoV, which are due to copying errors of its RNA genome, may destroy a weak immune system found in some individuals leading to FIP [6, 7]. The coronavirus spike (S) glycoprotein is a typical class 1 viral fusion protein and plays a central role in receptor binding and viral entry [8]. In addition to the S gene, there are several other genes in the FCoV genome. Nonstructural protein nsp12 encodes the RNA-dependent RNA polymerase protein, nsp13 encodes the helicase protein, and nsp 14 encodes the exoribonuclease protein, which are all essential for genome replication. The N gene encodes the nucleocapsid, which is commonly used in phylogenetic analysis [9]. The 7b gene is a small ORF that is located downstream of the N gene and is important for virulence [10].

Compared to the more predominant type I FCoV, type II FCoV viruses are only found in 2-30% of infections [11]. At present, a high incidence of type I FCoV occurs in Europe, Japan, Australia, Korea, and the USA.However, FIPV cases in Japan and Taiwan are more frequently associated with type II FCoV [5, 12], suggesting a difference in the geographical distribution of the different serotypes of FCoV. To date, almost all FCoV strains isolated from China are type I [13]. The goal of our study is to increase the sampling of FCoV in China and to also examine the selective pressures acting on the genes of these viruses isolated from different parts of the world. To study this, we obtained ascites samples from 31 cats with suspected FIP as well as feces samples from 8 healthy cats. These samples were collected at several pet hospitals in Liaoning Province, China, during the period October 2017 to May 2019. Of these 39 samples, 3 were found to be positive for FCoV. The adaptive evolutionary properties of FCoV, including selective pressure, were systematically analyzed using PAML and Datamonkey for the key FCoV functional proteins involved in viral entry, replication, and virulence. The aim of this study was to provide insight into the adaptive evolution of FCoV, which might provide insight into their pathogenic mechanisms.

## 2. Materials and Methods

### 2.1. Sampling

Samples were collected from veterinary hospitals in Liaoning Province, China, between October 2017 and May 2019. A total of 39 samples were obtained, with 31 being from cats with suspected FIP, as they had clinical symptoms such as loss of appetite and weight and increased abdominal girth with peritoneal effusion and/or pleural effusion [11] that are associated with this disease. Some, but not all, of the cases were examined for clinical hematologic and biochemical analysis. Effusions were collected by needle and syringe puncture guided by ultrasound. In addition, 8 fresh feces samples were collected from healthy cats using anal swabs, which were suspended in PBS and then stored at -80°C.

### 2.2. Viral RNA and Reverse Transcription

Viral RNA was extracted from 140 *μ*l of effusion or feces suspension with the QIAamp viral RNA minikit (Qiagen, Shenyang, China), following the manufacturer's instructions, and stored at -80°C. Extracted RNA was used as the template for cDNA synthesis with PrimeScript™ II 1st Strand cDNA Synthesis Kit (Takara, China) with random hexamers, following the manufacturer's instructions.

### 2.3. PCR, Cloning, and Sequencing

To amplify the S gene, we designed primers based on available feline coronavirus sequences. The partial S gene was divided into two segments, with primers designed separately for each part. The best primers and reaction conditions for each PCR reaction were selected using gradient PCR. Primer pairs S2B2-F and S2B2-R and SC1-F and SC1-R were used to amplify the 5′ and 3′ ends of the S gene, respectively. PCR was performed using 2 *μ*l of cDNA in a 10 *μ*l reaction containing 1 mmol/l concentration of each primer and 5 *μ*l of 2x Es Taq MasterMix (Beijing ComWin Biotech Co., Ltd.). To amplify partial nsp12, nsp13, and nsp14 gene sequences, we designed primer pairs 1b3F and 1b1R and 1b6F and 1b6R. To amplify part of the N gene, primer pair N1 and N2 was used [14]. To amplify the 7b gene, the previously designed primer pair 7b-F1 and 7b-R1 was used [3]. All PCR reactions had the same preheating temperature of 94°C and an extension temperature of 72°C. Annealing temperatures of the different amplifications are listed in Table 1. Products of the amplifications were separated by electrophoresis, with DNA from appropriate bands extracted, cloned, and sequenced (Sangon Biotech, Shanghai, China) to confirm virus detection. As previously described [15], type I FCoV is entirely a feline virus; however, type II FCoV is a recombinant of type I FCoV and a canine coronavirus (CCoV) that resulted in a FCoV genome containing the S gene and parts of the adjacent genes from CCoV. This results in the recombinant type II FCoV S gene having a different size from the type I S gene. Thus, amplification of the S gene allows typing of the FCoV virus.

### 2.4. Sequence Data and Phylogenetic Studies

In addition to the FCoV genomes sequenced in this study, genome sequences that contained all of the 5,895 bases that we amplified in our sequences and represent the diversity found in several select countries (United States, United Kingdom, Netherlands, and Belgium) were downloaded from the GenBank database for analysis. The accession numbers for these sequences are listed in Supporting Information Table S1. To establish the phylogenetic relationships of these viruses, conserved regions of the nsp12, nsp13, nsp14, S, 7b, and N genes were concatenated into a single sequence. From the 24 FCoV sequences from mainland China and other countries, an alignment of 5,895 bases was generated using ClustalW as implemented in Mega 6.0. Bayesian phylogenetic trees, based on the nucleotide sequences, which were constructed using MrBayes 3.1 with 5,000,000 generations, sampled every 100 generations, using the commands mcmcp samplefreq=100, a burnin of 20,000 generations, 4 chains, and the best-fit substitution model GTR+I+G applied, which had been selected using jModelTest [16]. Canine CoV (CCoV) sequences (accession numbers KC175339.1 and JQ404410.1) were used as the outgroup to root the trees.

### 2.5. Tests for Recombination

Since recombination can influence the detection of positive selection, we assessed whether recombination had occurred within our aligned dataset using the GARD (genetic algorithm for recombination detection) method [17]. A model selection procedure was run for each gene (the nsp12, nsp13, and nsp14 genes were considered to be a single gene), which sifts through all 203 possible time-reversible models in a hierarchical testing procedure combining nested LRT tests with AIC selection to pick a single “best-fitting” rate matrix, with site-to-site rate variation accounted for by the *β*–*Γ* distribution [17]. The best-fitting substitution models for the S gene was TrN model; however, there was no name of best model for the other three genes, and serial number of the best-fitting substitution models for nsp12-14, N, and 7b gene was (010230) with AIC of 20622.10, (010230) with AIC of 5874.10, and (012232) with AIC of 5108.58, respectively.

### 2.6. Evaluation of Selective Pressure

To detect the presence of positive selection in the FCoV sequences from the different countries, we applied the branch, site, and branch-site tests from the PAML suit [18]. Values of *ω* (the nonsynonymous/synonymous rate ratio) greater than 1 suggest positive selection. The *p* values can be calculated through likelihood ratio tests (LRT), where the null hypothesis would be rejected if the *p* value is <0.05 when the model allows positive selection. The branch model detects positive selection acting on particular lineages [19, 20]. A variety of models, including one ratio, free ratio, and two ratios (where the foreground lineages should be labeled), were analyzed. Comparing the free ratio and one ratio models examines whether the *ω* ratios differ among lineages. Comparing the two ratio and free ratio examines whether the *ω* ratios are different between the foreground and background lineages. The site models allow different *ω* ratios among sites [21, 22]. The one ratio model M0 assumes that the same *ω* exists for all sites across the phylogeny, while the nearly neutral model M1, positive selection model M2, discrete model M3, beta model M7, and beta and *ω* model M8 assume 2, 3, 3, 10, and 11 classes of codons, respectively, with different *ω* values, including some that suggest positive selection. If a site class with *ω* greater than one is found, which suggests positive selection, then sites with evidence of positive selection and a posterior probability *p* > 95% level were identified. Posterior probabilities were calculated by naïve empirical Bayes (NEB) and the Bayes empirical Bayes (BEB) [21]. Selection pressure was also analyzed through the Datamonkey suite of programs, including fixed effects likelihood (FEL; *p* < 0.05), random effects likelihood (REL; Bayes factor > 50), and mixed-effects model of evolution (MEME; *p* < 0.05). We considered a site to be under positive or negative selection when detected as such by at least two different methods.

## 3. Results

### 3.1. PCR Detection, Cloning, and Sequencing

Ascites samples were collected from 31 cats with suspected feline infectious peritonitis (FIP) along with feces samples from 8 healthy cats from pet hospitals in Liaoning Province, China, between October 2017 and May 2019. Of these samples, two of the suspected FIP cats (cat no. 7 FCoV China/LNS/2017/7I and no. 15 FCoV China/LNS/2017/15I) and one of the healthy cats (cat no. 3 FCoV China/LNS/2018/3E) were positive for FCoV. These three positive cats were derived from different households. All of the positive cases were classified as type I FCoV, as the amplified S gene products had a size that was expected for type I, rather than type II FCoV. Symptoms such as fever, anorexia, loss of weight, panting, and abdominal extension were observed in the suspected cases, and the two FIP-positive cats subsequently died within 10 days of admission to the hospital, while the healthy cat remained asymptomatic. Nsp12, nsp13, nsp14, S, 7b, and N gene clones were obtained from these three positive samples for sequencing and were used in the following analyses. Accession numbers for the FCoV sequences obtained in this study are provided in Supporting Information Table S2.

### 3.2. Phylogenetic Analysis

**Figure 1:**
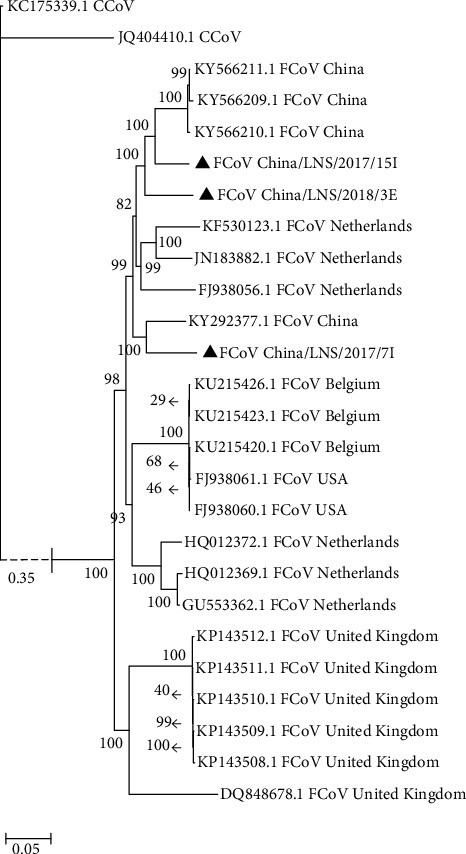
Phylogenetic analysis of the concatenated nsp12, nsp13, nsp14, S, 7b, and N gene sequences. The phylogenetic tree was reconstructed using the Bayesian method implemented in MrBayes 3.1. CCoV sequences were used as the outgroup for this analysis. Strains characterized in this study are labeled with triangles. Posterior probabilities are shown at the nodes of the phylogenetic tree. The branch to the CCoVs (outgroup) is shown with a dotted line, as it is very long and cannot be displayed at the same scale. Its branch length is 0.35 nucleotide substitutions per site and is indicated on the figure. Scale bar for the remaining branches is shown on the bottom length and represents 0.05 nucleotide substitutions per site.

An alignment of 5,895 bases, containing the partial nsp12, nsp13, nsp14, S, and N genes and the complete 7b gene sequence, was used for the phylogenetic analyses, with canine sequences used to root the tree. This analysis showed that our new FCoV sequences from mainland China clustered together previously characterized sequences from China, as well as a few sequences from the Netherlands as a distinct clade, separate from those of other countries (Figure 1). The analysis identified two additional clades, one composed of sequences only from the United Kingdom and a second composed of sequences from Belgium, United States, and the Netherlands (Figure 1). Apart from the clustering of the sequences from the United Kingdom, no clear geographic separation of FCoV sequences can be observed for the other two clades.

### 3.3. Detection of Recombination

Since recombinant sequences can lead to incorrect inference of positive selection [23], we tested our FCoV sequences for evidence of recombination using GARD with KH testing [17]. The results of this analysis for each gene is shown in Table 2. GARD detected evidence for one breakpoint within the nsp12-nsp14 genes and 2 within each of the S, N, and 7b genes. However, of these potential breakpoints, only those at locations 1020 (nsp12-nsp14), 360 and 582 (both N), and 434 (7b) were significant supported by the KH test, with *p* values < 0.05. As only a few recombination breakpoints were reliably detected by GARD, this suggests that recombination has little effect on our sequences and should not affect the detection of sites under positive selection [23].

### 3.4. Measurement of Selection Pressure

We used 5 methods to identify sites within the FCoV sequences with evidence for positive selection. The results of these analysis are shown in Table 3. For the nsp12-nsp14 gene region, four positively selected sites (6, 488, 562, and 631) were identified by at least two methods showing significance at *P* < 0.05, Bayes factors > 50, or posterior probabilities > 95% (Table 3). Site 562 in the nsp12-nsp14 genes showed evidence of positive selection with methods from both PAML and Datamonkey, while the three remaining sites in this gene fragment were only detected by two or more methods from Datamonkey. A total of twelve positively selected sites (22, 43, 101, 149, 151, 166, 167, 172, 175, 229, 245, and 475) were found in the S gene that were identified by at least two methods. Sites 151, 172, 175, and 245 showed evidence for positive selection from methods using both PAML and Datamonkey (Table 4), while the other eight sites were detected by methods from only one platform (either PAML or Datamonkey) (Table 4). For the N gene, only one positively selected site, position 21, was identified by methods from both PAML and Datamonkey, while three others (13, 52, and 195) were identified only with methods from PAML (Table 5). Three positively selected sites (41, 149, and 187) in the 7b gene were detected by methods from both PAML and Datamonkey (Table 6), with one site (5) showing evidence for positive selection only with methods from PAML (Table 6). In addition to positive selection, the FEL and REL methods can both identify sites with evidence of negative selection sites. Using these methods, we identified 106, 168, 25, and 17 negative selection sites in the nsp12-14, S, N, and 7b genes, respectively, which had significant evidence by both methods (Supporting Information Tables S3–S10). When the branch and branch-site models of PAML were applied to the alignments, no evidence for positive selection was found for any branch in the phylogenies.

## 4. Discussion

To study the evolution of feline CoV (FCoV) in China, we collected a total of 31 samples from cats, with 3 of these 31 samples testing positive for type I FCoV. The identification of type I FCoV in cats in Liaoning Province, China, is in line with a previous study that showed that type I FCoV is most prevalent in cats in China [13]. As the occurrence of FIP is associated with FECV, which results from FECV mutating into FIPV [24–26], we selected both FIPV and FECV sequences from several other countries for our phylogenetic analysis of our FCoV sequences. Phylogenetic analysis of the concatenated gene sequences obtained in this study yields some insight into the regionalism of type I FCoVs. All FCoV sequences from mainland China cluster together with a few sequences from the Netherlands. The FECV sequence of the asymptomatic cat (cat 3; FCoV China/LNS/2018/3E) clustered together with other FIPV sequences of China; however, it was an early diverging lineage in this clade (Figure 1); thus, the other FIPV sequences from China possibly contain additional disease-causing mutations. Sequences from the United Kingdom cluster together as a separate clade. The third cluster of FCoV sequences include isolates from Belgium, United States, and the Netherlands. Apart for the United Kingdom clade, an obvious geographical separation of the FCoV sequences is not observed, which might be a consequence of the trade in cats and tourism between countries. Our analysis shows that FCoV can be transmitted from one country to another, although a particular geographic position, such as the United Kingdom locates in an island, might limit its spread.

The Datamonkey suite of programs can identify codons under selection as well as the presence of recombinant sequences within a dataset [27]. Ideally, we should first determine whether recombination has occurred among the gene sequences, which can be assessed using GARD [17]. Although a breakpoint was identified within the nsp12-14 gene segment and 2 more within the other three genes examined, the number of breakpoints is less than three and thus likely would have little effect on the identification of sites experiencing positive selection by empirical Bayes methods [23]. In addition, it is known that the type II FCoV emerged via double recombination between type I FCoV and type II CCoV and that recombination events in type I FCoV have rarely been reported [28]. Previous studies have shown that the transmission of natural recombinant strain rarely occurs; thus, we should be cautious in concluding putative recombination event based on these computational analyses [29]. Although none of the branches of the FCoV phylogeny from different countries investigated here showed evidence of positive selection, when site models were applied, four sites in nsp12-14, ten sites in the S gene, 4 sites in the N gene, and 4 sites in the 7b gene were detected as having evidence for positive selection. Site 245 of the S gene, which previously had been shown to distinguishes FIPV from FECV [30], was found to be a positive selection site. Both the FEL and REL methods, from the Datamonkey suite of programs, can identify sites experiencing negative selection. From our analyses, 106, 168, 25, and 17 negative selection sites were identified by both FEL and REL in the nsp12-14, S, N, and 7b genes (Supporting Information Tables S3–S10). Viruses can experience both positive and negative selection. Positive selection leads to an increase in the abundance of a specific genetic variant, while negative selection results in genetic conservation [31–33]. A larger number of sites experiencing negative rather than positive selection were found for all of the genes that we analyzed, which suggests that most sites are conservative, and only a few can be adapted. The proteins encoded by the nsp12-14 genes are responsible for viral genome replication, with the S gene product responsible for viral entry into host cells, the N gene product responsible for the virus nucleocapsid protein, and the 7b gene product is responsible for viral virulence. Negative selection in these genes indicates that they are essential to the virus. The few positively selected changes that have occurred likely help these viruses survive and adapt under harmful conditions, with the change at site 245 of the S gene due to positive selection potentially related to the highly virulent FIPV of the virus [30]. Positive selection at other sites may contribute to the entry into host cells and enhanced virulence of viruses.

## 5. Conclusion

By analyzing the selective pressure experienced by genes in the FCoV genome involved in replication, entry, and virulence, we have identified a few sites that potentially experienced adaptive evolution. As negative selection occurs at a higher rate than positive selection within the FCoV genes, this suggests that only a few sites can beneficially adapt to allow greater infectivity in the host and that most sites are under strong negative selection to conserve function. A few of the positively selected sites in FCoV might be associated with the occurrence of FIPV and lead to these viruses causing a more fatal disease. Additional experimental analysis needs to be conducted to better understand the consequences of the positively selected changes identified here.

## Figures and Tables

**Table 1:** Primers for amplifying partial S, nsp 12, nsp 13, nsp 14, and N genes.

Primer	Amplified gene	Sequence (5′→3′)	Product size (bp)	Annealing temperature (°C)
S2B2-F	S	TTACAGCAGATGTTTTAAGG	1623	52
S2B2-R		CAGATTCAGGTTAAACCGGTTG		
SC1-F	S	TTGCGGTTTGTAATACTGGTG	1726	50
SC1-R		ACTTATGTAAAATGGCCTTGG		
1b3-F	nsp12-13	GAGTGGATTCAAACACCTGTAAC	1394	56.7
1b1-R		GTCAACCAAGAGAAGTACAATACCA		
1b6-F	nsp13-14	CTGTCAACCCTGAGTCAAAGC	1518	62.4
1b6-R		GGAATTGTAACGTAGACATGTACC		
N1	N	CCAAAAGACGTGATCGTTCTAA	664	50
N2		GTTTTCTTCCAGGTGTGTTTGT		

**Table 2:** KH testing of the significance of breakpoints identified by the GARD analysis.

				*p* value (Bonferroni corrected)	
Gene	Number	*△*AICc	Location	LHS	RHS
nsp12-nsp14	1	102.324	1020	0.0002 (0.0004)	0.0002 (0.0004)
S	2	52.739	360	0.0004 (0.0016)	0.0004 (0.0016)
			582	0.0004 (0.0016)	0.0004 (0.0016)
N	2	75.081	345	0.0016 (0.0064)	0.0164 (0.0656)
			526	0.0008 (0.0032)	0.0176 (0.0704)
7b	2	85.230	153	0.0148 (0.0592)	0.0004 (0.0016)
			434	0.0036 (0.0144)	0.0004 (0.0016)

Estimated breakpoint and KH test were conducted with nucleotide sequences. All *p* values were adjusted by Bonferroni correction.

**Table 3:** Positive selection sites for the nsp12, nsp13, and nsp14 gene sequences.

Position aa	FEL p^a^	REL bf^b^	MEME p^a^	M3 NEB pp^c^	M8 BEB pp^c^
6 A	0.035	418.130	0.018	-	-
488 A	-	67.086	0.002	-	-
562 I	0.016	727.473	0.020	0.988	-
631 T	-	57.722	0.042	-	-

aa: amino acid; ^a^*p* value; ^b^Bayes factor; ^c^posterior probability. Amino acids refer to sequence KY566211.1 FCoV China. “-” represents negative results of positive selection.

**Table 4:** Positive selection sites in the S gene.

Position aa	FEL p^a^	REL bf^b^	MEME p^a^	M3 NEB pp^c^	M8 BEB pp^c^
22 V	-	702.524	0.028	-	-
43 H	-	-	-	1.000	0.951
101 S	-	-	-	1.000	0.981
149 T	-	-	-	0.988	0.959
151 L	-	431.458	0.046	1.000	0.997
166 R	-	-	-	1.000	0.964
167 E	-	-	-	1.000	0.999
172 T	-	114.579	0.023	1.000	0.982
175 K	-	90.617	0.011	0.964	-
229 Q	-	302.459	0.0004	-	-
245 M	0.047	96.772	0.002	1.000	0.989
475 N	-	-	-	1.000	0.977

aa: amino acid; ^a^*p* value; ^b^Bayes factor; ^c^posterior probability. Amino acids refer to sequence KY566211.1 FCoV China. “-” represents negative results of positive selection.

**Table 5:** Positive selection sites in the N gene.

Position	FEL p^a^	REL bf^b^	MEME p^a^	M3 NEB pp^c^	M8 BEB pp^c^
13 Y	-	-	-	1.000	0.986
21 P	0.016	243.159	0.007	1.000	0.997
52 E	-	-	-	0.999	0.975
195 D	-	-	-	1.000	0.972

aa: amino acid. ^a^*p* value; ^b^Bayes factor; ^c^posterior probability. Amino acids refer to sequence KY566211.1 FCoV China. “-” represents negative results of positive selection.

**Table 6:** Positive selection sites in the 7b gene.

Position	FEL p^a^	REL bf^b^	MEME p^a^	M3 NEB pp^c^	M8 BEB pp^c^
5 F	—	—	—	0.999	0.975
41 H	0.006	333.652	0.0007	1.000	0.981
149 Y	—	166.322	—	0.995	0.960
187 T	—	—	0.012	0.999	0.972

aa: amino acid. ^a^*p* value; ^b^Bayes factor; ^c^posterior probability. Amino acids refer to sequence KY566211.1 FCoV China. “-” represents negative results of positive selection.

## Data Availability

All virus data obtained or analyzed during this study are included in this published article. Sequences of the obtained viruses have been uploaded in GenBank with accession numbers in Supplementary Materials Table S2.

## Supplementary Materials

Supplementary Materials Table S1: other coronavirus isolate sequences used in this study. Table S2: accession numbers of the coronavirus isolate sequences obtained in this study. Table S3: negative selection sites for the nsp12, nsp13, and nsp14 genes based on REL analysis. Table S4: negative selection sites for the S gene based on REL analysis. Table S5: negative selection sites for the N gene based on REL analysis. Table S6: negative selection sites for the 7b gene based on REL analysis. Table S7: negative selection sites for the nsp12, nsp13, and nsp14 genes based on FEL analysis. Table S8: negative selection sites for the S gene based on FEL analysis. Table S9: negative selection sites for the N gene based on FEL analysis. Table S10: negative selection sites for the 7b gene based on FEL analysis.

## References

[1] Barbara Regina Bank-Wolf, Stallkamp I., Wiese S., Moritz A., Tekes G., Thiel H.-J. (2014). Mutations of 3c and spike protein genes correlate with the occurrence of feline infectious peritonitis. *Veterinary Microbiology*.

[2] Hora A. S., Tonietti P. O., Taniwaki S. A. (2016). Feline coronavirus 3c protein: a candidate for a virulence marker?. *BioMed Research International*.

[3] Brown M. A., Troyer J. L., Pecon-Slattery J., Roelke M. E., O’Brien S. J. (2009). Genetics and pathogenesis of feline infectious peritonitis virus. *Emerging Infectious Diseases*.

[4] Terada Y., Shiozaki Y., Shimoda H. (2012). Feline infectious peritonitis virus with a large deletion in the 5′-terminal region of the spike gene retains its virulence for cats. *The Journal of General Virology*.

[5] Amer A., Suri A. S., Rahman O. A. (2012). Isolation and molecular characterization of type I and type II feline coronavirus in Malaysia. *Virology Journal*.

[6] Battilani M., Coradin T., Scagliarini A. (2003). Quasispecies composition and phylogenetic analysis of feline coronaviruses (FCoVs) in naturally infected cats. *FEMS Immunology and Medical Microbiology*.

[7] Herrewegh A. A. P. M., Mähler M., Hedrich H. J. (1997). Persistence and evolution of feline coronavirus in a closed cat-breeding colony. *Virology*.

[8] Lewis C. S., Porter E., Matthews D. (2015). Genotyping coronaviruses associated with feline infectious peritonitis. *The Journal of General Virology*.

[9] Woo P. C. Y., Huang Y., Lau S. K. P., Yuen K.-Y. (2010). Coronavirus genomics and bioinformatics analysis. *Viruses*.

[10] Vennema H., Rossen J. W. A., Wesseling J., Horzinek M. C., Rottier P. J. M. (1992). Genomic organization and expression of the 3' end of the canine and feline enteric coronaviruses. *Virology*.

[11] Wang Y. T., Su B. L., Hsieh L. E., Chueh L. L. (2013). An outbreak of feline infectious peritonitis in a Taiwanese shelter: epidemiologic and molecular evidence for horizontal transmission of a novel type II feline coronavirus. *Veterinary Research*.

[12] Duarte A., Veiga I., Tavares L. (2009). Genetic diversity and phylogenetic analysis of feline coronavirus sequences from Portugal. *Veterinary Microbiology*.

[13] Li C., Liu Q., Kong F. (2019). Circulation and genetic diversity of feline coronavirus type I and II from clinically healthy and FIP-suspected cats in China. *Transboundary and Emerging Diseases*.

[14] Yanli L., Jiangwei W. RT-PCR detection of feline infectious peritonitis virus.

[15] Addie D. D., Schaap I. A. T., Nicolson L., Jarrett O. (2003). Persistence and transmission of natural type I feline coronavirus infection. *The Journal of General Virology*.

[16] Ronquist F., Huelsenbeck J. P. (2003). MrBayes 3: Bayesian phylogenetic inference under mixed models. *Bioinformatics*.

[17] Kosakovsky Pond S. L., Posada D., Gravenor M. B., Woelk C. H., Frost S. D. W. (2006). Automated phylogenetic detection of recombination using a genetic algorithm. *Molecular Biology and Evolution*.

[18] Yang Z. (1997). PAML: a program package for phylogenetic analysis by maximum likelihood. *Computer Applications in the Biosciences*.

[19] Yang Z. (1998). Likelihood ratio tests for detecting positive selection and application to primate lysozyme evolution. *Molecular Biology and Evolution*.

[20] Yang Z., Nielsen R. (1998). Synonymous and nonsynonymous rate variation in nuclear genes of mammals. *Journal of Molecular Evolution*.

[21] Nielsen R., Yang Z. (1998). Likelihood models for detecting positively selected amino acid sites and applications to the HIV-1 envelope gene. *Genetics*.

[22] Yang Z. (2000). Maximum likelihood estimation on large phylogenies and analysis of adaptive evolution in human influenza virus A. *Journal of Molecular Evolution*.

[23] Anisimova M., Nielsen R., Yang Z. (2003). Effect of recombination on the accuracy of the likelihood method for detecting positive selection at amino acid sites. *Genetics*.

[24] Poland A. M., Vennema H., Foley J. E., Pedersen N. C. (1996). Two related strains of feline infectious peritonitis virus isolated from immunocompromised cats infected with a feline enteric coronavirus. *Journal of Clinical Microbiology*.

[25] Vennema H., Poland A., Foley J., Pedersen N. C. (1998). Feline infectious peritonitis viruses arise by mutation from endemic feline enteric coronaviruses. *Virology*.

[26] Pedersen N., Liu H., Dodd K., Pesavento P. (2009). Significance of coronavirus mutants in feces and diseased tissues of cats suffering from feline infectious peritonitis. *Viruses*.

[27] Poon A. F., Frost S. D., Pond S. L. (2009). Detecting signatures of selection from DNA sequences using Datamonkey. *Methods in Molecular Biology*.

[28] Lin C. N., Chang R. Y., Su B. L., Chueh L. L. (2013). Full genome analysis of a novel type II feline coronavirus NTU156. *Virus Genes*.

[29] Tang X., Li G., Vasilakis N. (2009). Differential stepwise evolution of SARS coronavirus functional proteins in different host species. *BMC Evolutionary Biology*.

[30] Chang H. W., Egberink H. F., Halpin R., Spiro D. J., Rottier P. J. M. (2012). Spike protein fusion peptide and feline coronavirus virulence. *Emerging Infectious Diseases*.

[31] Bush R. M. (2001). Predicting adaptive evolution. *Nature Reviews. Genetics*.

[32] Kosiol C., Bofkin L., Whelan S. (2006). Phylogenetics by likelihood: evolutionary modeling as a tool for understanding the genome. *Journal of Biomedical Informatics*.

[33] Yang Z., Nielsen R. (2002). Codon-substitution models for detecting molecular adaptation at individual sites along specific lineages. *Molecular Biology and Evolution*.

