# Supplementary material for: Adaptive Evolution of Feline Coronavirus Genes Based on Selection Analysis
Source: Biomed Res Int. 2020 Aug 13;2020:9089768. doi: 10.1155/2020/9089768 (PMC7453238; doi:10.1155/2020/9089768)
Supplement: Supplementary Materials — Table S1: other coronavirus isolate sequences used in this study. Table S2: accession numbers of the coronavirus isolate sequences obtained in this study. Table S3: negative selection sites for the nsp12, nsp13, and nsp14 genes based on REL analysis. Table S4: negative selection sites for the S gene based on REL analysis. Table S5: negative selection sites for the N gene based on REL analysis. Table S6: negative selection sites for the 7b gene based on REL analysis. Table S7: negative selection sites for the nsp12, nsp13, and nsp14 genes based on FEL analysis. Table S8: negative selection sites for the S gene based on FEL analysis. Table S9: negative selection sites for the N gene based on FEL analysis. Table S10: negative selection sites for the 7b gene based on FEL analysis. [file 9089768.f1.pdf]

**Table S1** Other Coronavirus isolate sequences used in this study.

| No. | Sequence | Collection date | Strain | Accession No. |
|---|---|---|---|---|
| 1 | genome | Nov-2016 | HLJ/HRB/2016/13 | KY566211.1 |
| 2 | genome | Nov-2016 | HLJ/HRB/2016/10 | KY566209.1 |
| 3 | genome | Oct-2016 | HLJ/DQ/2016/01 | KY292377.1 |
| 4 | genome | Nov-2016 | HLJ/HRB/2016/11 | KY566210.1 |
| 5 | genome | 2013 | Cat2_day84 | KU215426.1 |
| 6 | genome | 2013 | Cat3_day9 | KU215423.1 |
| 7 | genome | 2013 | Cat1_day7 | KU215420.1 |
| 8 | genome | Jan-2013 | 26M | KP143512.1 |
| 9 | genome | Jan-2013 | 80F | KP143511.1 |
| 10 | genome | Jan-2013 | 67F | KP143510.1 |
| 11 | genome | Jan-2013 | 65F | KP143509.1 |
| 12 | genome | Jan-2013 | 28O | KP143508.1 |
| 13 | genome | - | C1Je | DQ848678.1 |
| 14 | genome | Aug-2010 | Felis catus/NLD/UU88/2010 | KF530123.1 |
| 15 | genome | Jul-2010 | UU47 | JN183882.1 |
| 16 | genome | Sep-2007 | UU34 | HQ012372.1 |
| 17 | genome | Sep-2007 | UU21 | HQ012369.1 |
| 18 | genome | Sep-2007 | UU23 | GU553362.1 |
| 19 | genome | Mar-1998 | UU3 | FJ938061.1 |
| 20 | genome | Mar-1993 | UU2 | FJ938060.1 |
| 21 | genome | Mar-2007 | UU5 | FJ938056.1 |
| 22 | genome | - | TN-449 | JQ404410.1 |
| 23 | genome | 1971 | 171 | KC175339.1 |

"–" represents no definite collection date of the strain.

**Table S2** Accession numbers of the Coronavirus isolate sequences obtained in this study.

| No. | Sequence | Collection date | Strain | Accession No. |
|---|---|---|---|---|
| 1 | nsp12 | Oct-2017 | LNS/2017/7I | MN049959 |
| 2 | nsp13 | Oct-2017 | LNS/2017/7I | MN049962 |
| 3 | nsp14 | Oct-2017 | LNS/2017/7I | MN049965 |
| 4 | S | Oct-2017 | LNS/2017/7I | MN049968 |
| 5 | 7b | Oct-2017 | LNS/2017/7I | MN049974 |
| 6 | N | Oct-2017 | LNS/2017/7I | MN049971 |
| 7 | nsp12 | Dec-2017 | LNS/2017/15I | MN049960 |
| 8 | nsp13 | Dec-2017 | LNS/2017/15I | MN049963 |
| 9 | nsp14 | Dec-2017 | LNS/2017/15I | MN049966 |
| 10 | S | Dec-2017 | LNS/2017/15I | MN049969 |
| 11 | 7b | Dec-2017 | LNS/2017/15I | MN049975 |
| 12 | N | Dec-2017 | LNS/2017/15I | MN049972 |
| 13 | nsp12 | Apr-2018 | LNS/2018/3E | MN049961 |
| 14 | nsp13 | Apr-2018 | LNS/2018/3E | MN049964 |
| 15 | nsp14 | Apr-2018 | LNS/2018/3E | MN049967 |
| 16 | S | Apr-2018 | LNS/2018/3E | MN049970 |
| 17 | 7b | Apr-2018 | LNS/2018/3E | MN049976 |
| 18 | N | Apr-2018 | LNS/2018/3E | MN049973 |

**Table S3** Negative selection sites for the nsp12, nsp13, and nsp14 genes based on REL analysis.

| Codon | E[dS] | E[dN] | E[dN-dS] | Posterior Pr{dN>dS} | Bayes Factor {dN>dS} | Posterior Pr{dN<dS} | Bayes Factor {dN<dS} |
|---|---|---|---|---|---|---|---|
| 17 | 1.7262 | 0.014921 | -1.71128 | 0.000364243 | 0.010153 | 0.999636 | 98.4932 |
| 21 | 4.17433 | 0.153977 | -4.02035 | 0.000104159 | 0.0029026 | 0.999896 | 344.519 |
| 23 | 2.31418 | 0.018321 | -2.29586 | 0.000216034 | 0.0060209 | 0.999784 | 166.088 |
| 27 | 1.18117 | 0.016013 | -1.16516 | 7.49E-05 | 0.00208711 | 0.999925 | 479.13 |
| 28 | 3.32347 | 0.018396 | -3.30507 | 8.39E-05 | 0.00233729 | 0.999916 | 427.846 |
| 29 | 1.79067 | 0.017631 | -1.77304 | 4.45E-05 | 0.00123878 | 0.999956 | 807.245 |
| 37 | 2.86812 | 0.014243 | -2.85387 | 1.03E-05 | 0.000287868 | 0.99999 | 3473.82 |
| 47 | 2.12518 | 0.016012 | -2.10917 | 0.0002954 | 0.00823347 | 0.999705 | 121.455 |
| 65 | 4.4206 | 0.014072 | -4.40653 | 3.33E-08 | 9.27E-07 | 1 | 1079260 |
| 67 | 3.67002 | 0.01792 | -3.6521 | 3.77E-05 | 0.00105136 | 0.999962 | 951.153 |
| 70 | 2.0747 | 0.018602 | -2.0561 | 8.75E-05 | 0.00243742 | 0.999913 | 410.269 |
| 76 | 4.48 | 0.01601 | -4.46399 | 1.68E-08 | 4.69E-07 | 1 | 2133470 |
| 88 | 2.0334 | 0.018349 | -2.01506 | 0.000305249 | 0.00850806 | 0.999695 | 117.536 |
| 91 | 3.58377 | 0.016008 | -3.56776 | 1.62E-05 | 0.000451848 | 0.999984 | 2213.13 |

| | | | | | | | |
|---|---|---|---|---|---|---|---|
| 101 | 1.30468 | 0.013745 | -1.29093 | 0.000411464 | 0.0114698 | 0.999589 | 87.1856 |
| 107 | 2.67194 | 0.101979 | -2.56997 | 0.000365691 | 0.0101934 | 0.999634 | 98.1032 |
| 112 | 1.82932 | 0.017631 | -1.81169 | 0.000284618 | 0.00793286 | 0.999715 | 126.058 |
| 114 | 3.53179 | 0.012398 | -3.5194 | 8.21E-06 | 0.000228656 | 0.999992 | 4373.38 |
| 116 | 2.14649 | 0.012391 | -2.1341 | 8.95E-05 | 0.00249446 | 0.99991 | 400.889 |
| 118 | 1.80026 | 0.012398 | -1.78786 | 0.000128531 | 0.00358187 | 0.999871 | 279.184 |
| 134 | 1.74528 | 0.013734 | -1.73154 | 0.000162182 | 0.00451978 | 0.999838 | 221.25 |
| 135 | 1.37698 | 0.018455 | -1.35852 | 0.000259236 | 0.00722525 | 0.999741 | 138.403 |
| 145 | 1.24204 | 0.0132 | -1.22884 | 0.000460566 | 0.0128392 | 0.999539 | 77.8867 |
| 153 | 1.17501 | 0.014038 | -1.16098 | 0.000526735 | 0.0146847 | 0.999473 | 68.0981 |
| 157 | 2.20598 | 0.0124 | -2.19358 | 0.00015652 | 0.00436195 | 0.999843 | 229.255 |
| 168 | 1.17516 | 0.017071 | -1.15809 | 0.000547059 | 0.0152516 | 0.999453 | 65.5668 |
| 169 | 1.98923 | 0.014518 | -1.97471 | 0.000295698 | 0.00824177 | 0.999704 | 121.333 |
| 186 | 2.35515 | 0.013506 | -2.34164 | 0.000233213 | 0.00649978 | 0.999767 | 153.851 |
| 189 | 1.05294 | 0.016013 | -1.03693 | 0.000635349 | 0.0177147 | 0.999365 | 56.4505 |
| 192 | 1.42391 | 0.018458 | -1.40546 | 0.000168247 | 0.00468882 | 0.999832 | 213.273 |
| 198 | 3.20658 | 0.017799 | -3.18878 | 8.83E-05 | 0.00246123 | 0.999912 | 406.301 |
| 204 | 1.11787 | 0.014319 | -1.10355 | 0.000312723 | 0.00871646 | 0.999687 | 114.725 |
| 214 | 2.76452 | 0.016013 | -2.7485 | 3.20E-05 | 0.000890948 | 0.999968 | 1122.4 |
| 216 | 3.34601 | 0.022016 | -3.32399 | 0.00011163 | 0.00311082 | 0.999888 | 321.458 |
| 229 | 4.00268 | 0.016015 | -3.98666 | 1.38E-06 | 3.83E-05 | 0.999999 | 26089.4 |
| 237 | 2.07739 | 0.016205 | -2.06119 | 0.000199736 | 0.00556658 | 0.9998 | 179.644 |
| 239 | 2.03868 | 0.012401 | -2.02628 | 0.000127533 | 0.00355404 | 0.999872 | 281.37 |
| 240 | 1.63929 | 0.013736 | -1.62555 | 5.38E-05 | 0.00149978 | 0.999946 | 666.765 |
| 241 | 1.3623 | 0.018494 | -1.3438 | 0.000174018 | 0.00484969 | 0.999826 | 206.199 |
| 243 | 3.58406 | 0.013732 | -3.57033 | 1.21E-05 | 0.0003365 | 0.999988 | 2971.77 |
| 244 | 2.64029 | 0.018014 | -2.62228 | 0.000157799 | 0.00439763 | 0.999842 | 227.395 |
| 247 | 1.10964 | 0.017631 | -1.09201 | 0.00052389 | 0.0146053 | 0.999476 | 68.4681 |
| 263 | 2.93232 | 0.012388 | -2.91993 | 1.10E-05 | 0.000306235 | 0.999989 | 3265.47 |
| 290 | 3.41781 | 0.018385 | -3.39942 | 7.57E-05 | 0.00210959 | 0.999924 | 474.026 |
| 298 | 1.43173 | 0.012394 | -1.41933 | 0.000558224 | 0.0155631 | 0.999442 | 64.2547 |
| 307 | 2.64983 | 0.017411 | -2.63242 | 3.11E-05 | 0.000866439 | 0.999969 | 1154.15 |
| 313 | 2.44216 | 0.014072 | -2.42808 | 0.000106794 | 0.00297604 | 0.999893 | 336.017 |
| 317 | 3.13847 | 0.018415 | -3.12006 | 0.000100181 | 0.00279172 | 0.9999 | 358.203 |
| 328 | 1.85645 | 0.013695 | -1.84275 | 0.000147666 | 0.00411518 | 0.999852 | 243.002 |
| 334 | 3.83673 | 0.180961 | -3.65577 | 0.000655757 | 0.018284 | 0.999344 | 54.6925 |
| 336 | 2.22717 | 0.017631 | -2.20954 | 8.61E-05 | 0.00240026 | 0.999914 | 416.621 |
| 341 | 2.09574 | 0.019862 | -2.07587 | 8.73E-05 | 0.00243174 | 0.999913 | 411.228 |
| 373 | 2.24239 | 0.018022 | -2.22437 | 0.000293815 | 0.00818928 | 0.999706 | 122.111 |
| 390 | 2.97647 | 0.014098 | -2.96237 | 1.77E-05 | 0.000493571 | 0.999982 | 2026.05 |
| 399 | 2.82222 | 0.015629 | -2.80659 | 9.66E-05 | 0.00269287 | 0.999903 | 371.35 |
| 459 | 1.93134 | 0.016197 | -1.91514 | 0.000392935 | 0.0109531 | 0.999607 | 91.2986 |
| 477 | 1.54641 | 0.019894 | -1.52652 | 0.000153244 | 0.00427066 | 0.999847 | 234.156 |
| 485 | 4.38957 | 0.015989 | -4.37358 | 2.30E-07 | 6.40E-06 | 1 | 156284 |
| 486 | 2.47102 | 0.016126 | -2.45489 | 0.000172204 | 0.00479912 | 0.999828 | 208.372 |
| 495 | 3.67074 | 0.019833 | -3.65091 | 5.78E-05 | 0.00161189 | 0.999942 | 620.388 |
| 502 | 3.16559 | 0.019656 | -3.14593 | 0.000188436 | 0.00525158 | 0.999812 | 190.419 |
| 503 | 1.8901 | 0.019914 | -1.87019 | 3.89E-05 | 0.00108475 | 0.999961 | 921.871 |
| 507 | 2.32599 | 0.019656 | -2.30633 | 0.000349803 | 0.00975034 | 0.99965 | 102.561 |
| 508 | 3.83514 | 0.018022 | -3.81712 | 7.31E-06 | 0.000203704 | 0.999993 | 4909.08 |
| 515 | 1.18443 | 0.02106 | -1.16337 | 0.000218531 | 0.0060905 | 0.999781 | 164.19 |
| 517 | 3.71706 | 0.015629 | -3.70143 | 7.33E-06 | 0.000204137 | 0.999993 | 4898.67 |
| 523 | 4.36853 | 0.015989 | -4.35254 | 3.11E-07 | 8.66E-06 | 1 | 115430 |
| 532 | 3.35344 | 0.017311 | -3.33613 | 2.75E-05 | 0.000766947 | 0.999972 | 1303.87 |
| 536 | 4.07073 | 0.01563 | -4.05511 | 4.37E-06 | 0.000121777 | 0.999996 | 8211.73 |
| 537 | 1.24723 | 0.015073 | -1.23216 | 0.000426639 | 0.011893 | 0.999573 | 84.0832 |
| 558 | 2.86745 | 0.019365 | -2.84808 | 2.69E-05 | 0.000748563 | 0.999973 | 1335.89 |
| 560 | 1.29248 | 0.015861 | -1.27662 | 0.000130885 | 0.00364747 | 0.999869 | 274.162 |
| 590 | 4.51704 | 0.313235 | -4.2038 | 5.73E-08 | 1.60E-06 | 1 | 626543 |
| 601 | 4.03324 | 0.31741 | -3.71583 | 0.000255432 | 0.00711919 | 0.999745 | 140.465 |
| 606 | 3.9941 | 0.014088 | -3.98001 | 3.88E-06 | 0.000108156 | 0.999996 | 9245.86 |
| 607 | 2.7211 | 0.015989 | -2.70511 | 0.000107769 | 0.00300321 | 0.999892 | 332.977 |
| 610 | 4.25908 | 0.313695 | -3.94539 | 2.38E-05 | 0.000662502 | 0.999976 | 1509.43 |

| | | | | | | | |
|---|---|---|---|---|---|---|---|
| 618 | 2.4364 | 0.019755 | -2.41665 | 0.000319878 | 0.00891596 | 0.99968 | 112.158 |
| 619 | 3.26911 | 0.016145 | -3.25297 | 1.92E-05 | 0.000535655 | 0.999981 | 1866.87 |
| 624 | 3.84708 | 0.019366 | -3.82771 | 3.60E-06 | 0.000100408 | 0.999996 | 9959.33 |
| 630 | 2.55584 | 0.019365 | -2.53647 | 5.11E-05 | 0.00142386 | 0.999949 | 702.317 |
| 639 | 1.39766 | 0.018023 | -1.37963 | 0.000200221 | 0.0055801 | 0.9998 | 179.208 |
| 657 | 3.38112 | 0.020701 | -3.36042 | 2.51E-05 | 0.000699779 | 0.999975 | 1429.02 |
| 668 | 4.35324 | 0.018023 | -4.33522 | 4.57E-07 | 1.27E-05 | 1 | 78470.8 |
| 673 | 2.54402 | 0.019366 | -2.52466 | 0.000259166 | 0.00722329 | 0.999741 | 138.441 |
| 685 | 2.09369 | 0.019819 | -2.07388 | 0.000547155 | 0.0152543 | 0.999453 | 65.5553 |
| 688 | 2.44196 | 0.01563 | -2.42633 | 0.000194146 | 0.00541074 | 0.999806 | 184.817 |
| 692 | 1.61587 | 0.021154 | -1.59472 | 3.67E-05 | 0.00102355 | 0.999963 | 976.989 |
| 696 | 4.07522 | 0.016144 | -4.05907 | 5.30E-06 | 0.000147631 | 0.999995 | 6773.62 |
| 702 | 1.32563 | 0.0162 | -1.30943 | 0.000419704 | 0.0116996 | 0.99958 | 85.4732 |
| 718 | 4.39676 | 0.01715 | -4.37961 | 3.47E-07 | 9.67E-06 | 1 | 103385 |
| 722 | 3.73658 | 0.019836 | -3.71674 | 5.20E-05 | 0.0014494 | 0.999948 | 689.939 |
| 724 | 3.41094 | 0.018348 | -3.39259 | 7.38E-05 | 0.00205547 | 0.999926 | 486.506 |
| 733 | 2.59714 | 0.017338 | -2.5798 | 0.00019042 | 0.0053069 | 0.99981 | 188.434 |
| 735 | 2.92824 | 0.016394 | -2.91184 | 0.000417608 | 0.0116411 | 0.999582 | 85.9024 |
| 736 | 2.60064 | 0.01409 | -2.58655 | 8.92E-05 | 0.00248571 | 0.999911 | 402.299 |
| 739 | 4.37908 | 0.176515 | -4.20256 | 5.98E-06 | 0.000166623 | 0.999994 | 6001.57 |
| 743 | 2.54942 | 0.015059 | -2.53436 | 6.25E-06 | 0.000174263 | 0.999994 | 5738.45 |
| 748 | 4.04869 | 0.018208 | -4.03048 | 5.56E-06 | 0.000154952 | 0.999994 | 6453.61 |
| 760 | 1.21984 | 0.020522 | -1.19932 | 0.000374903 | 0.0104502 | 0.999625 | 95.6917 |
| 763 | 2.87498 | 0.015503 | -2.85948 | 9.07E-05 | 0.00252708 | 0.999909 | 395.713 |
| 765 | 1.10017 | 0.017441 | -1.08273 | 0.00032991 | 0.00919567 | 0.99967 | 108.747 |
| 767 | 3.15093 | 0.017312 | -3.13362 | 2.40E-05 | 0.000668997 | 0.999976 | 1494.78 |
| 799 | 2.85545 | 0.01563 | -2.83982 | 2.91E-05 | 0.00081181 | 0.999971 | 1231.81 |
| 800 | 1.57836 | 0.017741 | -1.56062 | 0.000246894 | 0.00688118 | 0.999753 | 145.324 |
| 804 | 4.39822 | 0.01546 | -4.38276 | 1.81E-07 | 5.04E-06 | 1 | 198528 |
| 819 | 1.12866 | 0.019656 | -1.109 | 0.000344291 | 0.00959665 | 0.999656 | 104.203 |
| 823 | 3.32754 | 0.014098 | -3.31344 | 1.57E-05 | 0.000438429 | 0.999984 | 2280.87 |
| 840 | 1.97504 | 0.01979 | -1.95525 | 0.000466517 | 0.0130051 | 0.999533 | 76.8927 |
| 853 | 4.51019 | 0.018019 | -4.49218 | 8.93E-09 | 2.49E-07 | 1 | 4016780 |
| 863 | 3.64559 | 0.017743 | -3.62785 | 1.80E-06 | 5.00E-05 | 0.999998 | 19982.8 |
| 874 | 3.69043 | 0.018024 | -3.67241 | 1.16E-05 | 0.000323113 | 0.999988 | 3094.89 |
| 881 | 3.0451 | 0.014094 | -3.031 | 1.85E-05 | 0.000514889 | 0.999982 | 1942.16 |
| 887 | 4.2864 | 0.020402 | -4.266 | 2.98E-06 | 8.30E-05 | 0.999997 | 12042.6 |
| 896 | 2.67655 | 0.018024 | -2.65853 | 0.000146953 | 0.00409532 | 0.999853 | 244.181 |
| 930 | 2.57223 | 0.01409 | -2.55814 | 9.20E-05 | 0.00256284 | 0.999908 | 390.192 |
| 935 | 3.32876 | 0.020351 | -3.3084 | 2.52E-05 | 0.000701087 | 0.999975 | 1426.36 |
| 941 | 1.14984 | 0.019953 | -1.12989 | 0.000378628 | 0.0105541 | 0.999621 | 94.75 |

**Table S4** Negative selection sites for the S gene based on REL analysis.

| Codon | E[dS] | E[dN] | E[dN-dS] | Posterior Pr{dN>dS} | Bayes Factor {dN>dS} | Posterior Pr{dN<dS} | Bayes Factor {dN<dS} |
|---|---|---|---|---|---|---|---|
| 3 | 1.82163 | 0.037776 | -1.78385 | 1.83E-05 | 0.000662 | 0.999982 | 1510.97 |
| 5 | 2.08896 | 0.026028 | -2.06293 | 2.06E-07 | 7.48E-06 | 1 | 133712 |
| 16 | 1.70057 | 0.234387 | -1.46619 | 0.000434 | 0.015752 | 0.999566 | 63.4849 |
| 19 | 1.55866 | 0.026727 | -1.53194 | 1.59E-05 | 0.000575 | 0.999984 | 1738.83 |
| 20 | 1.02738 | 0.026189 | -1.00119 | 0.000114 | 0.004138 | 0.999886 | 241.69 |
| 23 | 1.44976 | 0.02666 | -1.4231 | 6.36E-06 | 0.00023 | 0.999994 | 4340.65 |
| 24 | 2.58184 | 0.044951 | -2.53688 | 1.07E-08 | 3.89E-07 | 1 | 2571410 |
| 25 | 1.44259 | 0.028758 | -1.41383 | 7.57E-05 | 0.002745 | 0.999924 | 364.342 |
| 29 | 1.97955 | 0.026082 | -1.95347 | 1.42E-06 | 5.13E-05 | 0.999999 | 19486.5 |
| 35 | 1.4163 | 0.028413 | -1.38789 | 0.000155 | 0.005617 | 0.999845 | 178.025 |
| 42 | 1.27239 | 0.026187 | -1.2462 | 2.83E-05 | 0.001025 | 0.999972 | 975.607 |
| 52 | 2.07501 | 0.027498 | -2.04752 | 1.89E-06 | 6.86E-05 | 0.999998 | 14581.6 |
| 63 | 1.99412 | 0.202817 | -1.7913 | 1.57E-05 | 0.00057 | 0.999984 | 1753.14 |
| 66 | 1.43695 | 0.028766 | -1.40819 | 0.000164 | 0.005939 | 0.999836 | 168.379 |
| 69 | 1.83303 | 0.026188 | -1.80684 | 2.34E-06 | 8.49E-05 | 0.999998 | 11779.4 |
| 70 | 1.0653 | 0.026713 | -1.03859 | 6.49E-05 | 0.002353 | 0.999935 | 424.911 |
| 71 | 0.985939 | 0.026082 | -0.95986 | 0.000117 | 0.004255 | 0.999883 | 235.033 |
| 72 | 2.16642 | 0.026712 | -2.13971 | 2.60E-07 | 9.43E-06 | 1 | 106058 |
| 73 | 2.3002 | 0.031413 | -2.26879 | 2.35E-06 | 8.52E-05 | 0.999998 | 11731.6 |

| | | | | | | | |
|---|---|---|---|---|---|---|---|
| 74 | 0.922949 | 0.026082 | -0.89687 | 1.84E-05 | 0.000666 | 0.999982 | 1501.08 |
| 81 | 0.953071 | 0.026713 | -0.92636 | 1.41E-05 | 0.000509 | 0.999986 | 1963.55 |
| 88 | 1.25861 | 0.026728 | -1.23188 | 0.000145 | 0.005264 | 0.999855 | 189.972 |
| 89 | 1.10888 | 0.026082 | -1.0828 | 9.72E-06 | 0.000352 | 0.99999 | 2837.26 |
| 95 | 2.15268 | 0.027427 | -2.12525 | 4.25E-07 | 1.54E-05 | 1 | 64894.2 |
| 100 | 1.07798 | 0.026123 | -1.05186 | 3.82E-05 | 0.001384 | 0.999962 | 722.761 |
| 102 | 1.50233 | 0.028197 | -1.47413 | 2.57E-06 | 9.30E-05 | 0.999997 | 10752.8 |
| 103 | 1.85958 | 0.027987 | -1.8316 | 5.87E-07 | 2.13E-05 | 0.999999 | 46985.1 |
| 107 | 1.82359 | 0.026189 | -1.7974 | 1.38E-07 | 4.99E-06 | 1 | 200268 |
| 108 | 1.90854 | 0.026132 | -1.8824 | 1.45E-06 | 5.27E-05 | 0.999999 | 18967.6 |
| 111 | 1.91413 | 0.117894 | -1.79624 | 0.000454 | 0.016469 | 0.999546 | 60.7221 |
| 113 | 1.50095 | 0.026653 | -1.4743 | 2.96E-05 | 0.001074 | 0.99997 | 931.357 |
| 118 | 1.99894 | 0.028685 | -1.97026 | 1.01E-06 | 3.66E-05 | 0.999999 | 27306 |
| 122 | 1.33199 | 0.034853 | -1.29714 | 0.000314 | 0.01139 | 0.999686 | 87.7961 |
| 124 | 1.23629 | 0.02633 | -1.20996 | 9.26E-05 | 0.003356 | 0.999907 | 297.967 |
| 125 | 1.40434 | 0.025297 | -1.37905 | 6.14E-06 | 0.000222 | 0.999994 | 4494.7 |
| 126 | 1.05422 | 0.052601 | -1.00161 | 0.000132 | 0.004798 | 0.999868 | 208.399 |
| 132 | 1.49859 | 0.033475 | -1.46512 | 1.56E-05 | 0.000564 | 0.999984 | 1772.69 |
| 133 | 1.01492 | 0.025363 | -0.98955 | 1.06E-05 | 0.000385 | 0.999989 | 2598.17 |
| 137 | 0.955784 | 0.025478 | -0.93031 | 2.42E-05 | 0.000876 | 0.999976 | 1141.27 |
| 139 | 1.88951 | 0.052758 | -1.83675 | 4.06E-06 | 0.000147 | 0.999996 | 6799.7 |
| 145 | 1.27384 | 0.026327 | -1.24751 | 8.60E-05 | 0.003116 | 0.999914 | 320.896 |
| 150 | 1.79738 | 0.025364 | -1.77202 | 6.00E-07 | 2.17E-05 | 0.999999 | 46002.6 |
| 155 | 1.31262 | 0.026327 | -1.2863 | 8.08E-05 | 0.002929 | 0.999919 | 341.433 |
| 157 | 0.995633 | 0.047497 | -0.94814 | 0.000142 | 0.005148 | 0.999858 | 194.235 |
| 162 | 1.44454 | 0.025308 | -1.41923 | 2.22E-05 | 0.000805 | 0.999978 | 1242.93 |
| 163 | 1.57592 | 0.026293 | -1.54963 | 1.94E-05 | 0.000704 | 0.999981 | 1420.99 |
| 165 | 0.967217 | 0.04479 | -0.92243 | 2.94E-05 | 0.001065 | 0.999971 | 938.817 |
| 168 | 1.0302 | 0.026354 | -1.00385 | 7.65E-06 | 0.000277 | 0.999992 | 3604.25 |
| 169 | 0.942055 | 0.026386 | -0.91567 | 2.96E-05 | 0.001073 | 0.99997 | 931.861 |
| 170 | 1.03371 | 0.02521 | -1.0085 | 4.64E-06 | 0.000168 | 0.999995 | 5941.29 |
| 176 | 1.52238 | 0.035388 | -1.48699 | 4.06E-05 | 0.001473 | 0.999959 | 678.726 |
| 177 | 1.40999 | 0.025283 | -1.38471 | 5.74E-06 | 0.000208 | 0.999994 | 4804.12 |
| 182 | 0.953657 | 0.026472 | -0.92718 | 7.61E-06 | 0.000276 | 0.999992 | 3623.85 |
| 185 | 1.81446 | 0.048979 | -1.76548 | 1.76E-05 | 0.000637 | 0.999982 | 1569.38 |
| 186 | 2.00172 | 0.026518 | -1.9752 | 5.43E-06 | 0.000197 | 0.999995 | 5080.62 |
| 187 | 0.917326 | 0.025566 | -0.89176 | 2.65E-05 | 0.000961 | 0.999973 | 1040.9 |
| 189 | 0.881004 | 0.025364 | -0.85564 | 3.42E-05 | 0.001239 | 0.999966 | 807.269 |
| 191 | 1.03911 | 0.025824 | -1.01329 | 1.45E-05 | 0.000526 | 0.999985 | 1901.23 |
| 192 | 1.15607 | 0.025525 | -1.13054 | 0.000163 | 0.005899 | 0.999837 | 169.523 |
| 195 | 1.65393 | 0.027084 | -1.62684 | 1.72E-05 | 0.000623 | 0.999983 | 1603.88 |
| 199 | 1.36842 | 0.029374 | -1.33905 | 8.68E-05 | 0.003146 | 0.999913 | 317.906 |
| 202 | 1.41863 | 0.027114 | -1.39152 | 2.68E-05 | 0.000973 | 0.999973 | 1028.2 |
| 203 | 2.40444 | 0.026418 | -2.37802 | 7.45E-08 | 2.70E-06 | 1 | 370196 |
| 205 | 1.70027 | 0.027896 | -1.67237 | 5.32E-06 | 0.000193 | 0.999995 | 5181.44 |
| 206 | 2.00698 | 0.026477 | -1.9805 | 1.31E-06 | 4.74E-05 | 0.999999 | 21082.6 |
| 207 | 1.48847 | 0.028921 | -1.45955 | 6.12E-05 | 0.002218 | 0.999939 | 450.758 |
| 208 | 2.2098 | 0.027081 | -2.18272 | 8.33E-07 | 3.02E-05 | 0.999999 | 33117 |
| 209 | 1.60052 | 0.026351 | -1.57417 | 1.57E-05 | 0.000568 | 0.999984 | 1761.88 |
| 210 | 1.28434 | 0.028922 | -1.25541 | 2.50E-05 | 0.000906 | 0.999975 | 1103.65 |
| 211 | 2.44975 | 0.02843 | -2.42132 | 7.87E-08 | 2.85E-06 | 1 | 350387 |
| 225 | 1.10375 | 0.027879 | -1.07587 | 1.51E-05 | 0.000546 | 0.999985 | 1832.82 |
| 226 | 1.78179 | 0.059486 | -1.7223 | 7.68E-05 | 0.002783 | 0.999923 | 359.307 |
| 227 | 1.32935 | 0.027083 | -1.30227 | 2.81E-05 | 0.001019 | 0.999972 | 980.894 |
| 228 | 1.30601 | 0.02892 | -1.2771 | 4.65E-05 | 0.001687 | 0.999953 | 592.673 |
| 230 | 1.65262 | 0.029297 | -1.62332 | 1.00E-05 | 0.000364 | 0.99999 | 2750.42 |
| 235 | 1.52746 | 0.029374 | -1.49809 | 4.08E-06 | 0.000148 | 0.999996 | 6762.88 |
| 236 | 2.0808 | 0.026477 | -2.05432 | 3.56E-07 | 1.29E-05 | 1 | 77564.8 |
| 238 | 2.59857 | 0.026264 | -2.57231 | 1.77E-09 | 6.40E-08 | 1 | 15621300 |
| 241 | 2.63753 | 0.028091 | -2.60944 | 3.85E-10 | 1.40E-08 | 1 | 71684300 |
| 244 | 1.22348 | 0.026351 | -1.19713 | 2.85E-05 | 0.001031 | 0.999972 | 969.576 |
| 246 | 1.40743 | 0.027896 | -1.37954 | 5.11E-05 | 0.001853 | 0.999949 | 539.589 |
| 249 | 1.42097 | 0.026478 | -1.3945 | 1.66E-05 | 0.000603 | 0.999983 | 1657.59 |

| | | | | | | | |
|---|---|---|---|---|---|---|---|
| 251 | 1.34188 | 0.026351 | -1.31553 | 2.60E-05 | 0.000943 | 0.999974 | 1060.66 |
| 252 | 2.50037 | 0.027081 | -2.47329 | 1.38E-08 | 5.00E-07 | 1 | 1999120 |
| 253 | 1.78987 | 0.026351 | -1.76352 | 2.20E-06 | 7.96E-05 | 0.999998 | 12563.9 |
| 254 | 2.08795 | 0.027083 | -2.06087 | 5.03E-07 | 1.82E-05 | 0.999999 | 54874.6 |
| 255 | 1.44529 | 0.025915 | -1.41937 | 1.27E-05 | 0.00046 | 0.999987 | 2175.06 |
| 260 | 1.44618 | 0.027083 | -1.4191 | 5.80E-06 | 0.00021 | 0.999994 | 4754.29 |
| 262 | 2.27212 | 0.026351 | -2.24577 | 1.55E-07 | 5.63E-06 | 1 | 177648 |
| 263 | 1.61265 | 0.027426 | -1.58522 | 1.97E-05 | 0.000714 | 0.99998 | 1401.32 |
| 265 | 1.9882 | 0.029298 | -1.95891 | 1.75E-06 | 6.34E-05 | 0.999998 | 15775.8 |
| 266 | 1.76011 | 0.029374 | -1.73073 | 3.71E-05 | 0.001346 | 0.999963 | 743.105 |
| 267 | 1.40472 | 0.027084 | -1.37764 | 3.51E-05 | 0.001271 | 0.999965 | 786.773 |
| 269 | 2.40049 | 0.02891 | -2.37158 | 7.31E-08 | 2.65E-06 | 1 | 377331 |
| 270 | 1.64568 | 0.032234 | -1.61345 | 0.000198 | 0.007174 | 0.999802 | 139.4 |
| 271 | 1.47383 | 0.026473 | -1.44736 | 2.12E-05 | 0.000767 | 0.999979 | 1303.88 |
| 273 | 1.53273 | 0.027084 | -1.50565 | 7.67E-06 | 0.000278 | 0.999992 | 3596.3 |
| 274 | 1.99067 | 0.029107 | -1.96156 | 5.09E-07 | 1.85E-05 | 0.999999 | 54182.5 |
| 284 | 1.14292 | 0.026351 | -1.11657 | 9.71E-06 | 0.000352 | 0.99999 | 2841.6 |
| 292 | 1.22885 | 0.027024 | -1.20183 | 4.59E-05 | 0.001665 | 0.999954 | 600.714 |
| 295 | 1.56015 | 0.026352 | -1.5338 | 2.91E-06 | 0.000106 | 0.999997 | 9471.41 |
| 298 | 1.92056 | 0.029378 | -1.89118 | 1.98E-05 | 0.000719 | 0.99998 | 1390.91 |
| 299 | 1.14001 | 0.027084 | -1.11293 | 6.65E-05 | 0.002411 | 0.999933 | 414.838 |
| 309 | 2.12154 | 0.028127 | -2.09341 | 2.44E-06 | 8.84E-05 | 0.999998 | 11314.3 |
| 311 | 1.66744 | 0.029297 | -1.63815 | 4.01E-05 | 0.001452 | 0.99996 | 688.72 |
| 317 | 2.45446 | 0.028386 | -2.42608 | 3.28E-07 | 1.19E-05 | 1 | 84068 |
| 319 | 2.13352 | 0.029383 | -2.10413 | 2.82E-06 | 0.000102 | 0.999997 | 9776.1 |
| 323 | 1.84293 | 0.027082 | -1.81585 | 2.58E-06 | 9.36E-05 | 0.999997 | 10684.1 |
| 330 | 1.24601 | 0.026351 | -1.21966 | 2.12E-05 | 0.000768 | 0.999979 | 1301.28 |
| 340 | 2.05655 | 0.092423 | -1.96413 | 0.000213 | 0.007717 | 0.999787 | 129.588 |
| 341 | 1.44896 | 0.029295 | -1.41967 | 0.000281 | 0.010192 | 0.999719 | 98.121 |
| 348 | 1.39914 | 0.026128 | -1.37301 | 4.20E-06 | 0.000152 | 0.999996 | 6572.7 |
| 350 | 1.47662 | 0.047551 | -1.42907 | 0.000355 | 0.012885 | 0.999645 | 77.6107 |
| 352 | 0.978867 | 0.027024 | -0.95184 | 3.79E-05 | 0.001375 | 0.999962 | 727.454 |
| 358 | 1.95576 | 0.029382 | -1.92638 | 1.74E-05 | 0.00063 | 0.999983 | 1586.53 |
| 359 | 1.57418 | 0.05854 | -1.51564 | 0.000346 | 0.012548 | 0.999654 | 79.6914 |
| 363 | 2.02392 | 0.026351 | -1.99757 | 1.68E-06 | 6.07E-05 | 0.999998 | 16466.9 |
| 367 | 1.45348 | 0.026942 | -1.42653 | 6.70E-07 | 2.43E-05 | 0.999999 | 41195.6 |
| 371 | 2.45919 | 0.02791 | -2.43128 | 3.07E-08 | 1.11E-06 | 1 | 899759 |
| 372 | 2.4796 | 0.027738 | -2.45186 | 9.17E-08 | 3.33E-06 | 1 | 300749 |
| 373 | 1.38004 | 0.028717 | -1.35133 | 4.34E-06 | 0.000157 | 0.999996 | 6362.23 |
| 378 | 1.44066 | 0.026351 | -1.41431 | 5.08E-06 | 0.000184 | 0.999995 | 5435.64 |
| 392 | 1.31518 | 0.026351 | -1.28883 | 2.93E-05 | 0.001061 | 0.999971 | 942.863 |
| 393 | 1.45508 | 0.027338 | -1.42774 | 0.000132 | 0.004781 | 0.999868 | 209.143 |
| 394 | 2.27369 | 0.027467 | -2.24622 | 9.14E-07 | 3.31E-05 | 0.999999 | 30194.3 |
| 396 | 2.13747 | 0.028915 | -2.10855 | 1.72E-07 | 6.22E-06 | 1 | 160671 |
| 397 | 1.06587 | 0.026351 | -1.03952 | 0.000117 | 0.004236 | 0.999883 | 236.093 |
| 399 | 1.72763 | 0.028996 | -1.69863 | 2.61E-05 | 0.000948 | 0.999974 | 1055.33 |
| 405 | 2.3747 | 0.027084 | -2.34761 | 3.74E-08 | 1.35E-06 | 1 | 738373 |
| 408 | 1.83015 | 0.03223 | -1.79792 | 3.74E-05 | 0.001355 | 0.999963 | 738.149 |
| 409 | 1.83522 | 0.026353 | -1.80887 | 1.79E-06 | 6.47E-05 | 0.999998 | 15450.2 |
| 416 | 1.84776 | 0.028242 | -1.81952 | 4.23E-06 | 0.000153 | 0.999996 | 6527.24 |
| 419 | 2.37625 | 0.026477 | -2.34978 | 3.53E-08 | 1.28E-06 | 1 | 782354 |
| 423 | 1.72087 | 0.02644 | -1.69443 | 7.50E-06 | 0.000272 | 0.999992 | 3678.12 |
| 424 | 1.86619 | 0.02651 | -1.83968 | 2.80E-06 | 0.000102 | 0.999997 | 9848.18 |
| 432 | 2.16508 | 0.028907 | -2.13617 | 2.85E-07 | 1.03E-05 | 1 | 96937.6 |
| 437 | 1.38092 | 0.026476 | -1.35445 | 1.79E-05 | 0.000649 | 0.999982 | 1540.12 |
| 438 | 2.5834 | 0.02708 | -2.55632 | 1.96E-09 | 7.09E-08 | 1 | 14096400 |
| 439 | 2.58907 | 0.028889 | -2.56018 | 7.28E-08 | 2.64E-06 | 1 | 378729 |
| 441 | 1.49046 | 0.025916 | -1.46455 | 2.92E-06 | 0.000106 | 0.999997 | 9445.58 |
| 442 | 1.3272 | 0.026476 | -1.30072 | 2.03E-05 | 0.000734 | 0.99998 | 1361.68 |
| 446 | 2.59214 | 0.089518 | -2.50263 | 6.66E-07 | 2.41E-05 | 0.999999 | 41417 |
| 458 | 2.33482 | 0.026475 | -2.30835 | 1.02E-07 | 3.71E-06 | 1 | 269717 |
| 465 | 1.93318 | 0.028921 | -1.90426 | 4.00E-07 | 1.45E-05 | 1 | 69020.5 |
| 469 | 1.48108 | 0.026128 | -1.45495 | 3.58E-06 | 0.00013 | 0.999996 | 7697.57 |

| | | | | | | | |
|---|---|---|---|---|---|---|---|
| 471 | 1.59831 | 0.026289 | -1.57202 | 1.33E-05 | 0.000481 | 0.999987 | 2077.67 |
| 474 | 1.31023 | 0.09499 | -1.21524 | 0.000169 | 0.00612 | 0.999831 | 163.389 |
| 479 | 2.20177 | 0.316551 | -1.88522 | 0.000167 | 0.006058 | 0.999833 | 165.078 |
| 480 | 1.61566 | 0.026477 | -1.58918 | 1.68E-05 | 0.000609 | 0.999983 | 1641.44 |
| 483 | 1.85419 | 0.029297 | -1.82489 | 8.12E-06 | 0.000294 | 0.999992 | 3398.04 |
| 487 | 1.42667 | 0.025916 | -1.40075 | 1.55E-05 | 0.00056 | 0.999985 | 1785.44 |
| 496 | 1.59323 | 0.027084 | -1.56615 | 4.44E-06 | 0.000161 | 0.999996 | 6219.28 |
| 500 | 1.3376 | 0.027338 | -1.31026 | 0.000175 | 0.006354 | 0.999825 | 157.375 |
| 505 | 1.3277 | 0.026289 | -1.30141 | 0.0001 | 0.003634 | 0.9999 | 275.145 |
| 529 | 1.64204 | 0.028921 | -1.61312 | 4.66E-05 | 0.001688 | 0.999953 | 592.465 |
| 531 | 1.86455 | 0.027023 | -1.83753 | 0.000326 | 0.011829 | 0.999674 | 84.5375 |
| 533 | 2.02256 | 0.128209 | -1.89435 | 0.000401 | 0.014528 | 0.999599 | 68.8337 |
| 547 | 1.59031 | 0.089367 | -1.50094 | 2.95E-05 | 0.001069 | 0.999971 | 935.693 |
| 550 | 2.1004 | 0.026287 | -2.07412 | 8.72E-07 | 3.16E-05 | 0.999999 | 31651.3 |
| 564 | 2.63921 | 0.028852 | -2.61036 | 1.90E-08 | 6.90E-07 | 1 | 1450040 |
| 565 | 2.08242 | 0.028995 | -2.05342 | 1.11E-05 | 0.000404 | 0.999989 | 2478.16 |
| 566 | 2.56839 | 0.032228 | -2.53617 | 1.66E-07 | 6.02E-06 | 1 | 166222 |
| 568 | 2.21409 | 0.026351 | -2.18774 | 9.62E-07 | 3.49E-05 | 0.999999 | 28672.3 |
| 571 | 1.15766 | 0.026399 | -1.13126 | 9.54E-07 | 3.46E-05 | 0.999999 | 28917.3 |
| 574 | 2.4711 | 0.056727 | -2.41438 | 1.75E-06 | 6.34E-05 | 0.999998 | 15784.3 |
| 577 | 2.16139 | 0.087272 | -2.07412 | 0.000139 | 0.005056 | 0.999861 | 197.804 |
| 578 | 1.06906 | 0.029287 | -1.03977 | 1.11E-05 | 0.000404 | 0.999989 | 2475.86 |
| 579 | 1.87363 | 0.032224 | -1.8414 | 3.37E-05 | 0.001222 | 0.999966 | 818.21 |

**Table S5** Negative selection sites for the N gene based on REL analysis.

| Codon | E[dS] | E[dN] | E[dN-dS] | Posterior Pr{dN>dS} | Bayes Factor {dN>dS} | Posterior Pr{dN<dS} | Bayes Factor {dN<dS} |
|---|---|---|---|---|---|---|---|
| 12 | 2.0865 | 0.066751 | -2.01975 | 1.12E-05 | 0.000371 | 0.999989 | 2694.72 |
| 27 | 2.09613 | 0.252606 | -1.84352 | 0.000307 | 0.010133 | 0.999693 | 98.6898 |
| 40 | 1.76896 | 0.068472 | -1.70049 | 4.43E-05 | 0.001463 | 0.999956 | 683.474 |
| 51 | 2.13794 | 0.082036 | -2.0559 | 2.93E-05 | 0.000968 | 0.999971 | 1033.57 |
| 53 | 2.18592 | 0.067497 | -2.11843 | 9.06E-07 | 2.99E-05 | 0.999999 | 33417.6 |
| 59 | 2.14994 | 0.070723 | -2.07922 | 5.96E-06 | 0.000197 | 0.999994 | 5085.6 |
| 63 | 2.17091 | 0.251353 | -1.91955 | 7.27E-05 | 0.0024 | 0.999927 | 416.585 |
| 67 | 2.09921 | 0.069689 | -2.02952 | 1.21E-05 | 0.000399 | 0.999988 | 2504.81 |
| 78 | 2.17466 | 0.354745 | -1.81992 | 0.000361 | 0.011935 | 0.999639 | 83.784 |
| 83 | 2.13353 | 0.052564 | -2.08096 | 5.82E-07 | 1.92E-05 | 0.999999 | 52012.6 |
| 86 | 2.16872 | 0.275502 | -1.89322 | 0.000432 | 0.014269 | 0.999568 | 70.0832 |
| 88 | 2.08449 | 0.066949 | -2.01754 | 9.49E-06 | 0.000313 | 0.999991 | 3192 |
| 95 | 2.19103 | 0.25893 | -1.9321 | 2.31E-05 | 0.000761 | 0.999977 | 1313.86 |
| 97 | 2.19306 | 0.072132 | -2.12093 | 5.47E-07 | 1.80E-05 | 0.999999 | 55423 |
| 100 | 2.15146 | 0.255905 | -1.89556 | 0.000159 | 0.005252 | 0.999841 | 190.406 |
| 102 | 2.07872 | 0.06532 | -2.0134 | 8.71E-06 | 0.000288 | 0.999991 | 3477.96 |
| 105 | 2.14759 | 0.064871 | -2.08272 | 7.51E-05 | 0.002479 | 0.999925 | 403.445 |
| 125 | 2.16213 | 0.234356 | -1.92777 | 4.07E-05 | 0.001343 | 0.999959 | 744.597 |
| 127 | 1.77259 | 0.046391 | -1.7262 | 1.11E-06 | 3.67E-05 | 0.999999 | 27236.7 |
| 142 | 2.18234 | 0.225612 | -1.95673 | 9.28E-06 | 0.000306 | 0.999991 | 3264.53 |
| 187 | 1.77722 | 0.05387 | -1.72335 | 3.44E-05 | 0.001136 | 0.999966 | 879.984 |
| 190 | 1.74326 | 0.045741 | -1.69752 | 2.13E-06 | 7.02E-05 | 0.999998 | 14241.6 |
| 192 | 2.17192 | 0.046444 | -2.12548 | 1.58E-07 | 5.22E-06 | 1 | 191682 |
| 198 | 1.93619 | 0.045821 | -1.89037 | 1.26E-06 | 4.16E-05 | 0.999999 | 24030.7 |
| 206 | 1.86426 | 0.220063 | -1.6442 | 0.000271 | 0.008956 | 0.999729 | 111.655 |

**Table S6** Negative selection sites for the 7b gene based on REL analysis.

| Codon | E[dS] | E[dN] | E[dN-dS] | Posterior Pr{dN>dS} | Bayes Factor {dN>dS} | Posterior Pr{dN<dS} | Bayes Factor {dN<dS} |
|---|---|---|---|---|---|---|---|
| 18 | 2.85236 | 0.274583 | -2.57778 | 0.005708 | 0.014717 | 0.994292 | 67.9499 |
| 31 | 2.77147 | 0.106709 | -2.66476 | 0.000161 | 0.000412 | 0.999839 | 2424.88 |
| 35 | 2.82677 | 0.13741 | -2.68936 | 0.004111 | 0.010583 | 0.995889 | 94.4899 |
| 38 | 1.76589 | 0.111773 | -1.65411 | 0.002621 | 0.006737 | 0.997379 | 148.43 |
| 48 | 2.81801 | 0.112317 | -2.70569 | 0.000309 | 0.000793 | 0.999691 | 1260.27 |
| 51 | 2.84076 | 0.136994 | -2.70377 | 0.004168 | 0.010732 | 0.995832 | 93.184 |
| 56 | 2.52257 | 0.150482 | -2.37209 | 0.007436 | 0.019206 | 0.992564 | 52.0667 |
| 57 | 2.9287 | 0.262381 | -2.66632 | 0.001347 | 0.003457 | 0.998653 | 289.245 |
| 63 | 2.71157 | 0.139316 | -2.57226 | 0.003012 | 0.007744 | 0.996988 | 129.129 |

| | | | | | | | |
|---|---|---|---|---|---|---|---|
| 72 | 2.2328 | 0.125927 | -2.10687 | 0.005087 | 0.013108 | 0.994913 | 76.2911 |
| 81 | 2.93572 | 0.139484 | -2.79624 | 0.000145 | 0.000373 | 0.999855 | 2680.8 |
| 90 | 2.49364 | 0.148409 | -2.34523 | 0.007554 | 0.019514 | 0.992446 | 51.2449 |
| 109 | 2.81808 | 0.147907 | -2.67017 | 0.002115 | 0.005433 | 0.997885 | 184.067 |
| 111 | 2.72739 | 0.142309 | -2.58508 | 0.003062 | 0.007874 | 0.996938 | 126.997 |
| 132 | 2.57992 | 0.13315 | -2.44677 | 0.003689 | 0.009493 | 0.996311 | 105.344 |
| 141 | 2.63907 | 0.142332 | -2.49673 | 0.004336 | 0.011165 | 0.995664 | 89.5646 |
| 168 | 2.22101 | 0.115292 | -2.10572 | 0.002373 | 0.006099 | 0.997627 | 163.961 |

**Table S7** Negative selection sites for the nsp12, nsp13, and nsp14 genes based on FEL analysis.

| Codon | dS | dN | dN Leaves | dN/dS | Normalized dN-dS | dS (when dN=dS) | Log(L) | LRT | P value |
|---|---|---|---|---|---|---|---|---|---|
| 17 | 3.80295 | 0 | 0 | 0 | -7.68616 | 1.23139 | -13.1625 | 4.92463 | 0.026477 |
| 21 | 29.175 | 0.871957 | 0 | 0.03 | -57.2034 | 3.94255 | -20.0692 | 9.58784 | 0.001959 |
| 23 | 5.49586 | 0 | 0 | 0 | -11.1077 | 1.9232 | -9.63202 | 5.17807 | 0.022874 |
| 27 | 2.40463 | 0 | 0 | 0 | -4.86 | 1.48359 | -12.794 | 4.2082 | 0.040229 |
| 28 | 10.4752 | 0 | 0 | 0 | -21.1716 | 2.38519 | -10.1301 | 7.36015 | 0.006669 |
| 29 | 3.41701 | 0 | 0 | 0 | -6.90613 | 1.81562 | -12.58 | 4.49809 | 0.033933 |
| 37 | 5.75732 | 0 | 0 | 0 | -11.6362 | 1.99952 | -11.6144 | 8.05862 | 0.004529 |
| 47 | 5.36919 | 0 | 0 | 0 | -10.8517 | 1.4668 | -13.61 | 5.5392 | 0.018595 |
| 65 | 11.6527 | 0 | 0 | 0 | -23.5514 | 3.1196 | -18.4733 | 13.9653 | 0.000186 |
| 67 | 9.51868 | 0 | 0 | 0 | -19.2383 | 2.61886 | -10.8 | 8.27736 | 0.004014 |
| 70 | 4.4065 | 0 | 0 | 0 | -8.90599 | 2.12115 | -16.7142 | 4.77257 | 0.028917 |
| 76 | 14.3658 | 0 | 0 | 0 | -29.0349 | 4.31164 | -20.5747 | 13.5286 | 0.000235 |
| 88 | 4.68708 | 0 | 0 | 0 | -9.47308 | 1.74044 | -10.6614 | 4.58603 | 0.032234 |
| 91 | 10.7706 | 0 | 0 | 0 | -21.7685 | 2.28985 | -14.924 | 8.99482 | 0.002707 |
| 101 | 2.51973 | 0 | 0 | 0 | -5.09264 | 1.05925 | -18.574 | 4.69325 | 0.030281 |
| 107 | 5.24137 | 0.553082 | 0 | 0.106 | -9.47553 | 2.04201 | -26.1877 | 4.90429 | 0.02679 |
| 112 | 3.821 | 0 | 0 | 0 | -7.72264 | 1.78554 | -10.9459 | 4.74274 | 0.029422 |
| 114 | 15.3754 | 0 | 0 | 0 | -31.0754 | 1.58672 | -16.6588 | 11.6419 | 0.000645 |
| 116 | 4.8195 | 0 | 0 | 0 | -9.74071 | 1.22698 | -14.1048 | 7.84572 | 0.005094 |
| 118 | 3.93061 | 0 | 0 | 0 | -7.94418 | 1.13965 | -13.3437 | 6.85185 | 0.008855 |
| 134 | 4.00879 | 0 | 0 | 0 | -8.10219 | 1.33811 | -13.4315 | 6.48963 | 0.010851 |
| 145 | 2.36569 | 0 | 0 | 0 | -4.7813 | 0.966826 | -11.4419 | 4.61772 | 0.031643 |
| 153 | 2.41391 | 0 | 0 | 0 | -4.87877 | 1.03251 | -14.902 | 4.58117 | 0.032325 |
| 157 | 10.3359 | 0 | 0 | 0 | -20.8899 | 1.04107 | -15.3557 | 7.20964 | 0.007251 |
| 169 | 5.58928 | 0 | 0 | 0 | -11.2965 | 1.2985 | -15.7562 | 5.28038 | 0.021567 |
| 186 | 8.68031 | 0 | 0 | 0 | -17.5438 | 1.09357 | -21.3992 | 6.86435 | 0.008793 |
| 192 | 3.1534 | 0 | 0 | 0 | -6.37336 | 1.78215 | -15.63 | 3.96408 | 0.046481 |
| 198 | 9.00342 | 0 | 0 | 0 | -18.1969 | 2.21658 | -13.3865 | 7.28092 | 0.006969 |
| 204 | 1.89732 | 0 | 0 | 0 | -3.83469 | 1.10023 | -12.2152 | 3.9504 | 0.04686 |
| 214 | 14.385 | 0 | 0 | 0 | -29.0736 | 2.05059 | -19.5336 | 7.14636 | 0.007512 |
| 216 | 11.3811 | 0 | 0 | 0 | -23.0023 | 3.08298 | -11.4461 | 5.95342 | 0.014689 |
| 229 | 8.99039 | 0 | 0 | 0 | -18.1705 | 2.88099 | -18.0036 | 9.59835 | 0.001948 |
| 237 | 4.65076 | 0 | 0 | 0 | -9.39967 | 1.62043 | -15.1865 | 5.92253 | 0.014948 |
| 239 | 4.68468 | 0 | 0 | 0 | -9.46823 | 1.12623 | -12.1855 | 7.37774 | 0.006604 |
| 240 | 3.57956 | 0 | 0 | 0 | -7.23466 | 1.48564 | -12.8483 | 6.40364 | 0.011389 |
| 241 | 3.08936 | 0 | 0 | 0 | -6.24392 | 1.77412 | -16.1086 | 3.91776 | 0.047779 |
| 243 | 15.2815 | 0 | 0 | 0 | -30.8856 | 1.77033 | -18.6021 | 10.7106 | 0.001065 |
| 244 | 6.20815 | 0 | 0 | 0 | -12.5473 | 2.03269 | -11.422 | 5.97697 | 0.014494 |
| 263 | 5.68928 | 0 | 0 | 0 | -11.4986 | 1.62462 | -15.8515 | 9.76301 | 0.001781 |
| 290 | 15.8478 | 0 | 0 | 0 | -32.0301 | 2.40676 | -10.5512 | 7.94153 | 0.004831 |
| 298 | 3.42332 | 0 | 0 | 0 | -6.91889 | 0.844523 | -13.8648 | 5.66243 | 0.017332 |
| 307 | 6.174 | 0 | 0 | 0 | -12.4783 | 2.07173 | -16.9172 | 6.12128 | 0.013356 |
| 313 | 5.54221 | 0 | 0 | 0 | -11.2014 | 1.48665 | -15.8726 | 7.38591 | 0.006574 |
| 317 | 8.04785 | 0 | 0 | 0 | -16.2656 | 2.32391 | -9.45089 | 6.77488 | 0.009245 |
| 328 | 4.21547 | 0 | 0 | 0 | -8.51991 | 1.36771 | -12.5625 | 6.66143 | 0.009852 |
| 334 | 19.8412 | 1.13361 | 0 | 0.057 | -37.8099 | 3.88558 | -17.0196 | 5.22653 | 0.022245 |
| 336 | 4.98591 | 0 | 0 | 0 | -10.077 | 1.90125 | -10.9474 | 5.24366 | 0.022027 |
| 341 | 4.23825 | 0 | 0 | 0 | -8.56595 | 2.23521 | -12.2692 | 4.26397 | 0.038929 |
| 373 | 14.0363 | 0 | 0 | 0 | -28.3689 | 1.80305 | -15.1214 | 4.92688 | 0.026442 |
| 390 | 5.76221 | 0 | 0 | 0 | -11.646 | 1.95123 | -13.6841 | 8.38581 | 0.003782 |
| 399 | 6.54983 | 0 | 0 | 0 | -13.2379 | 1.85061 | -14.7686 | 7.18411 | 0.007355 |
| 459 | 4.49196 | 0 | 0 | 0 | -9.07873 | 1.45223 | -12.9764 | 4.93999 | 0.026242 |

| | | | | | | | | | |
|---|---|---|---|---|---|---|---|---|---|
| 485 | 14.9142 | 0 | 0 | 0 | -30.1433 | 3.55195 | -18.6649 | 12.7486 | 0.000356 |
| 486 | 7.45578 | 0 | 0 | 0 | -15.0689 | 1.70953 | -10.2581 | 6.11307 | 0.013419 |
| 495 | 13.5154 | 0 | 0 | 0 | -27.316 | 3.02532 | -11.2985 | 7.69428 | 0.00554 |
| 502 | 105.889 | 0 | 0 | 0 | -214.013 | 2.2373 | -12.4934 | 10.6755 | 0.001086 |
| 503 | 3.70132 | 0 | 0 | 0 | -7.48076 | 2.16918 | -14.6954 | 3.93641 | 0.047252 |
| 507 | 7.07987 | 0 | 0 | 0 | -14.3092 | 1.96679 | -10.1718 | 4.58103 | 0.032328 |
| 508 | 7.85727 | 0 | 0 | 0 | -15.8804 | 3.2006 | -17.2924 | 7.71049 | 0.00549 |
| 517 | 7.59197 | 0 | 0 | 0 | -15.3442 | 2.51294 | -17.8716 | 8.95682 | 0.002764 |
| 523 | 14.3169 | 0 | 0 | 0 | -28.936 | 3.46232 | -19.9278 | 12.4973 | 0.000408 |
| 532 | 8.83182 | 0 | 0 | 0 | -17.85 | 2.4244 | -12.595 | 7.4752 | 0.006255 |
| 536 | 13.8924 | 0 | 0 | 0 | -28.078 | 2.64593 | -17.0823 | 11.1214 | 0.000853 |
| 537 | 2.65043 | 0 | 0 | 0 | -5.35679 | 1.25639 | -16.9031 | 4.36146 | 0.036761 |
| 558 | 5.33628 | 0 | 0 | 0 | -10.7852 | 2.70017 | -12.5707 | 5.54149 | 0.018571 |
| 560 | 2.77355 | 0 | 0 | 0 | -5.60564 | 1.51082 | -19.8736 | 4.55263 | 0.032868 |
| 590 | 26.7669 | 0.902817 | 0.718612 | 0.034 | -52.274 | 5.77643 | -32.7358 | 10.6015 | 0.00113 |
| 606 | 11.4567 | 0 | 0 | 0 | -23.1552 | 2.27228 | -17.6468 | 11.4041 | 0.000733 |
| 607 | 6.13424 | 0 | 0 | 0 | -12.3979 | 1.89917 | -14.4007 | 6.83668 | 0.00893 |
| 610 | 9.91743 | 0 | 1.57786 | 0 | -20.0442 | 3.90417 | -26.4713 | 10.1096 | 0.001475 |
| 618 | 8.41444 | 0 | 0 | 0 | -17.0065 | 1.99296 | -9.85601 | 4.84437 | 0.027737 |
| 619 | 8.07092 | 0 | 0 | 0 | -16.3122 | 2.29694 | -11.7161 | 7.69868 | 0.005526 |
| 624 | 9.12584 | 0 | 0 | 0 | -18.4443 | 3.20953 | -16.1972 | 7.30111 | 0.006891 |
| 630 | 5.16527 | 0 | 0 | 0 | -10.4396 | 2.44342 | -12.6044 | 5.17459 | 0.02292 |
| 639 | 3.16432 | 0 | 0 | 0 | -6.39542 | 1.8214 | -16.4567 | 4.07147 | 0.043613 |
| 657 | 6.09628 | 0 | 0 | 0 | -12.3212 | 3.23152 | -20.2154 | 4.06824 | 0.043697 |
| 668 | 37.7353 | 0 | 0 | 0 | -76.267 | 4.12968 | -19.4373 | 13.7902 | 0.000204 |
| 673 | 11.2469 | 0 | 0 | 0 | -22.7312 | 2.0238 | -15.1599 | 5.28234 | 0.021543 |
| 685 | 5.9525 | 0 | 0 | 0 | -12.0306 | 1.7468 | -10.9158 | 4.04893 | 0.044199 |
| 688 | 6.01187 | 0 | 0 | 0 | -12.1506 | 1.5579 | -12.1842 | 6.39007 | 0.011476 |
| 696 | 20.5445 | 0 | 0 | 0 | -41.5226 | 2.95672 | -13.5213 | 10.7428 | 0.001047 |
| 702 | 2.8218 | 0 | 0 | 0 | -5.70315 | 1.40635 | -11.3965 | 4.12644 | 0.042218 |
| 718 | 42.371 | 0 | 0 | 0 | -85.6362 | 4.11627 | -15.3103 | 14.3538 | 0.000151 |
| 722 | 16.1123 | 0 | 0 | 0 | -32.5646 | 3.09033 | -11.0541 | 8.09562 | 0.004437 |
| 724 | 10.7749 | 0 | 0 | 0 | -21.7772 | 2.45065 | -12.8261 | 7.50968 | 0.006137 |
| 733 | 7.25486 | 0 | 0 | 0 | -14.6628 | 1.86627 | -11.3754 | 5.99025 | 0.014385 |
| 735 | 10.7001 | 0 | 0 | 0 | -21.6261 | 1.59293 | -11.2056 | 7.35207 | 0.006699 |
| 736 | 5.86055 | 0 | 0 | 0 | -11.8448 | 1.58397 | -14.6648 | 7.53119 | 0.006064 |
| 739 | 12.0838 | 0 | 0.971258 | 0 | -24.4227 | 4.21842 | -22.1686 | 9.91479 | 0.00164 |
| 743 | 4.61247 | 0 | 0 | 0 | -9.32229 | 2.16559 | -22.488 | 7.1659 | 0.00743 |
| 748 | 10.7079 | 0 | 0 | 0 | -21.6419 | 3.16341 | -13.5994 | 8.97602 | 0.002735 |
| 763 | 6.82407 | 0 | 0 | 0 | -13.7922 | 1.87424 | -17.0733 | 7.29562 | 0.006912 |
| 767 | 6.63923 | 0 | 0 | 0 | -13.4186 | 2.48797 | -11.8398 | 6.83528 | 0.008937 |
| 799 | 6.3887 | 0 | 0 | 0 | -12.9122 | 2.0125 | -15.0351 | 7.17483 | 0.007393 |
| 800 | 3.49344 | 0 | 0 | 0 | -7.06062 | 1.79868 | -12.5421 | 4.46056 | 0.034686 |
| 804 | 13.6506 | 0 | 0 | 0 | -27.5894 | 3.58426 | -21.608 | 12.6631 | 0.000373 |
| 823 | 8.8512 | 0 | 0 | 0 | -17.8892 | 1.86092 | -15.6805 | 9.13772 | 0.002504 |
| 840 | 4.63951 | 0 | 0 | 0 | -9.37694 | 1.81684 | -10.5595 | 3.88897 | 0.048604 |
| 853 | 32.0055 | 0 | 0 | 0 | -64.6865 | 5.99112 | -20.7986 | 14.7775 | 0.000121 |
| 863 | 19.3601 | 0 | 0 | 0 | -39.1288 | 2.61644 | -15.2728 | 8.43667 | 0.003677 |
| 874 | 8.24063 | 0 | 0 | 0 | -16.6552 | 2.99853 | -15.8874 | 7.5277 | 0.006076 |
| 881 | 6.90857 | 0 | 0 | 0 | -13.963 | 1.85491 | -14.9269 | 8.40234 | 0.003747 |
| 887 | 70.1445 | 0 | 0 | 0 | -141.77 | 4.41226 | -11.8751 | 12.4168 | 0.000425 |
| 896 | 6.12674 | 0 | 0 | 0 | -12.3828 | 2.21045 | -15.2315 | 5.82281 | 0.01582 |
| 930 | 5.77156 | 0 | 0 | 0 | -11.6649 | 1.57679 | -15.7991 | 7.49127 | 0.0062 |
| 935 | 8.80705 | 0 | 0 | 0 | -17.8 | 2.95065 | -10.8045 | 5.90536 | 0.015095 |

**Table S8** Negative selection sites for the S gene based on FEL analysis.

| Codon | dS | dN | dN Leaves | dN/dS | Normalize dN-dS | dS (when dN=dS) | Log(L) | LRT | P value |
|---|---|---|---|---|---|---|---|---|---|
| 3 | 3.5841 | 0 | 0.2697 | 0 | -4.18161 | 1.69829 | -24.6954 | 6.73196 | 0.00947 |
| 5 | 5.45648 | 0 | 0 | 0 | -6.36615 | 1.34146 | -12.9895 | 9.90628 | 0.001647 |
| 16 | 3.2305 | 0 | 0.676593 | 0 | -3.76906 | 1.13899 | -26.2815 | 7.74379 | 0.00539 |
| 19 | 3.38701 | 0 | 0 | 0 | -3.95167 | 0.971781 | -12.3616 | 6.17448 | 0.012961 |
| 20 | 1.58071 | 0 | 0 | 0 | -1.84423 | 0.568044 | -12.9585 | 4.02465 | 0.04484 |
| 23 | 2.65069 | 0 | 0 | 0 | -3.09259 | 1.06369 | -11.9515 | 6.13227 | 0.013274 |

| | | | | | | | | | |
|---|---|---|---|---|---|---|---|---|---|
| 24 | 9.19026 | 0 | 0.314907 | 0 | -10.7224 | 2.16664 | -29.5063 | 14.7696 | 0.000121 |
| 25 | 2.96788 | 0 | 0 | 0 | -3.46266 | 0.989204 | -11.2814 | 4.46538 | 0.034588 |
| 29 | 7.10802 | 0 | 0 | 0 | -8.29303 | 1.1185 | -16.5097 | 9.50679 | 0.002047 |
| 35 | 2.92972 | 0 | 0 | 0 | -3.41814 | 0.87744 | -9.31782 | 4.48125 | 0.034269 |
| 42 | 2.15958 | 0 | 0 | 0 | -2.51961 | 0.753145 | -13.5157 | 5.18232 | 0.022818 |
| 52 | 7.00299 | 0 | 0 | 0 | -8.17048 | 1.44138 | -18.1958 | 8.63317 | 0.003301 |
| 63 | 3.8025 | 0 | 0.62108 | 0 | -4.43643 | 1.35725 | -28.6217 | 9.51786 | 0.002035 |
| 66 | 3.02854 | 0 | 0 | 0 | -3.53344 | 0.913673 | -8.94106 | 4.46961 | 0.034503 |
| 69 | 4.49128 | 0 | 0 | 0 | -5.24004 | 1.08461 | -15.0431 | 8.18466 | 0.004225 |
| 70 | 1.61113 | 0 | 0 | 0 | -1.87972 | 0.744638 | -11.9003 | 4.15711 | 0.04146 |
| 71 | 1.45992 | 0 | 0 | 0 | -1.70331 | 0.541464 | -12.6659 | 3.90076 | 0.048264 |
| 72 | 4.30306 | 0 | 0 | 0 | -5.02044 | 1.58615 | -17.7997 | 9.18709 | 0.002437 |
| 73 | 8.42824 | 0 | 0 | 0 | -9.83335 | 2.22102 | -13.5059 | 8.0093 | 0.004654 |
| 74 | 1.36231 | 0 | 0 | 0 | -1.58943 | 0.767158 | -13.1008 | 4.57302 | 0.032479 |
| 81 | 1.37391 | 0 | 0 | 0 | -1.60296 | 0.859013 | -15.0975 | 4.20376 | 0.040335 |
| 88 | 2.06169 | 0 | 0 | 0 | -2.4054 | 0.605564 | -10.2809 | 3.91936 | 0.047733 |
| 89 | 1.86485 | 0 | 0 | 0 | -2.17574 | 0.858914 | -13.2155 | 5.51818 | 0.01882 |
| 95 | 4.63592 | 0 | 0 | 0 | -5.40879 | 1.62185 | -17.8958 | 8.55185 | 0.003452 |
| 100 | 1.66327 | 0 | 0 | 0 | -1.94056 | 0.704732 | -12.658 | 4.66499 | 0.030784 |
| 102 | 2.66113 | 0 | 0 | 0 | -3.10478 | 1.43987 | -21.053 | 5.74392 | 0.016546 |
| 103 | 3.47683 | 0 | 0 | 0 | -4.05646 | 1.64154 | -23.2584 | 6.47874 | 0.010917 |
| 107 | 3.28321 | 0 | 0 | 0 | -3.83057 | 1.35486 | -16.1683 | 8.35868 | 0.003839 |
| 108 | 4.15656 | 0 | 0 | 0 | -4.84952 | 1.1659 | -17.0062 | 8.68092 | 0.003216 |
| 111 | 5.2961 | 0 | 0.607981 | 0 | -6.17903 | 1.82036 | -17.6679 | 5.58568 | 0.018108 |
| 113 | 3.8444 | 0 | 0 | 0 | -4.48531 | 0.842549 | -12.712 | 5.64751 | 0.01748 |
| 118 | 5.68957 | 0 | 0 | 0 | -6.63809 | 1.63367 | -11.944 | 7.09431 | 0.007733 |
| 122 | 2.40139 | 0 | 0.259888 | 0 | -2.80174 | 0.627515 | -20.2872 | 5.54336 | 0.018551 |
| 124 | 2.13545 | 0 | 0 | 0 | -2.49145 | 0.642188 | -9.18799 | 4.38359 | 0.036287 |
| 125 | 2.95788 | 0 | 0 | 0 | -3.451 | 0.58165 | -18.8078 | 6.49712 | 0.010805 |
| 126 | 1.81437 | 0 | 0.349754 | 0 | -2.11685 | 1.01322 | -21.4159 | 5.17542 | 0.022909 |
| 132 | 2.80182 | 0 | 0.235798 | 0 | -3.26892 | 0.965439 | -18.9221 | 7.47296 | 0.006263 |
| 133 | 1.60566 | 0 | 0 | 0 | -1.87334 | 0.612622 | -14.5858 | 5.57389 | 0.01823 |
| 137 | 1.23011 | 0 | 0 | 0 | -1.43519 | 0.579787 | -13.4807 | 4.42192 | 0.03548 |
| 139 | 3.0989 | 0 | 0.35088 | 0 | -3.61553 | 1.52633 | -25.2236 | 8.13364 | 0.004345 |
| 145 | 2.26972 | 0 | 0 | 0 | -2.64811 | 0.64966 | -9.97292 | 4.48383 | 0.034217 |
| 150 | 3.97691 | 0 | 0 | 0 | -4.63992 | 0.911851 | -17.0693 | 9.42897 | 0.002136 |
| 155 | 2.43934 | 0 | 0 | 0 | -2.84601 | 0.655095 | -10.0239 | 4.6087 | 0.03181 |
| 157 | 1.68259 | 0 | 0.315545 | 0 | -1.9631 | 0.959033 | -24.9871 | 4.95774 | 0.025974 |
| 162 | 16.1621 | 0 | 0 | 0 | -18.8566 | 0.410124 | -10.0458 | 6.48025 | 0.010908 |
| 163 | 4.5913 | 0 | 0 | 0 | -5.35673 | 0.840844 | -10.122 | 6.17009 | 0.012993 |
| 165 | 1.68497 | 0 | 0.28893 | 0 | -1.96588 | 1.06129 | -24.498 | 5.11078 | 0.023778 |
| 168 | 1.59859 | 0 | 0 | 0 | -1.86509 | 0.909511 | -21.0274 | 4.76227 | 0.02909 |
| 169 | 1.41157 | 0 | 0 | 0 | -1.6469 | 0.798349 | -14.8912 | 4.19771 | 0.040479 |
| 170 | 1.64132 | 0 | 0 | 0 | -1.91495 | 0.559178 | -15.1482 | 6.16237 | 0.01305 |
| 176 | 2.81726 | 0 | 0.249662 | 0 | -3.28693 | 1.00066 | -22.2325 | 7.38557 | 0.006575 |
| 177 | 3.18845 | 0 | 0 | 0 | -3.72001 | 0.583651 | -15.5988 | 6.60752 | 0.010155 |
| 182 | 1.54818 | 0 | 0 | 0 | -1.80628 | 0.952901 | -17.0461 | 4.63989 | 0.031237 |
| 185 | 4.91586 | 0 | 0.318472 | 0 | -5.7354 | 1.26347 | -15.2934 | 7.51254 | 0.006127 |
| 186 | 7.75014 | 0 | 0 | 0 | -9.04219 | 1.1785 | -12.0102 | 8.8176 | 0.002983 |
| 187 | 1.35066 | 0 | 0 | 0 | -1.57583 | 0.618491 | -12.5345 | 4.66554 | 0.030774 |
| 189 | 0.995219 | 0 | 0 | 0 | -1.16114 | 0.495618 | -13.3499 | 4.06499 | 0.043781 |
| 191 | 1.62225 | 0 | 0 | 0 | -1.8927 | 0.776865 | -13.7356 | 5.06471 | 0.024418 |
| 192 | 1.73143 | 0 | 0 | 0 | -2.02008 | 0.554088 | -13.1736 | 5.31378 | 0.021157 |
| 195 | 3.43314 | 0 | 0 | 0 | -4.00549 | 1.10167 | -16.5151 | 6.31815 | 0.011951 |
| 199 | 2.53117 | 0 | 0 | 0 | -2.95315 | 1.06911 | -11.925 | 4.04469 | 0.044311 |
| 202 | 2.62954 | 0 | 0 | 0 | -3.06792 | 0.995874 | -13.4014 | 5.32794 | 0.020986 |
| 203 | 6.81377 | 0 | 0 | 0 | -7.94972 | 1.76529 | -18.7734 | 11.5065 | 0.000694 |
| 205 | 3.12995 | 0 | 0 | 0 | -3.65176 | 1.40833 | -16.3713 | 6.22419 | 0.012602 |
| 206 | 4.38935 | 0 | 0 | 0 | -5.12111 | 1.34396 | -16.7541 | 8.53315 | 0.003487 |
| 207 | 3.17575 | 0 | 0 | 0 | -3.70518 | 1.08355 | -10.2103 | 4.53603 | 0.033188 |
| 208 | 5.09669 | 0 | 0 | 0 | -5.94637 | 1.65468 | -17.0542 | 9.03411 | 0.00265 |
| 209 | 5.64663 | 0 | 0 | 0 | -6.588 | 0.898688 | -15.068 | 6.44217 | 0.011144 |
| 210 | 2.25703 | 0 | 0 | 0 | -2.63331 | 1.18538 | -12.068 | 4.32392 | 0.03758 |

| | | | | | | | | | | |
|---|---|---|---|---|---|---|---|---|---|---|
| 211 | 5.34976 | 0 | 0 | 0 | -6.24164 | 2.3553 | -24.7629 | 8.6128 | 0.003338 |
| 225 | 1.92727 | 0 | 0 | 0 | -2.24857 | 1.11994 | -14.6949 | 4.48436 | 0.034206 |
| 226 | 3.571 | 0 | 0.365845 | 0 | -4.16634 | 1.3605 | -19.4543 | 6.75579 | 0.009344 |
| 227 | 2.44259 | 0 | 0 | 0 | -2.8498 | 1.00445 | -13.6829 | 5.23478 | 0.02214 |
| 228 | 2.2208 | 0 | 0 | 0 | -2.59104 | 1.13483 | -12.7697 | 4.16735 | 0.04121 |
| 230 | 3.1607 | 0 | 0 | 0 | -3.68763 | 1.45462 | -11.8975 | 5.27179 | 0.021674 |
| 235 | 2.69599 | 0 | 0 | 0 | -3.14544 | 1.44717 | -13.3582 | 4.85216 | 0.027612 |
| 236 | 4.37981 | 0 | 0 | 0 | -5.10998 | 1.46048 | -16.8802 | 8.86274 | 0.002911 |
| 238 | 15.0849 | 0 | 0 | 0 | -17.5998 | 2.2971 | -20.4162 | 18.2718 | 1.92E-05 |
| 241 | 13.2458 | 0 | 0 | 0 | -15.454 | 3.3337 | -23.0837 | 16.3283 | 5.33E-05 |
| 244 | 2.13967 | 0 | 0 | 0 | -2.49638 | 0.836436 | -13.9062 | 5.20967 | 0.022462 |
| 246 | 2.73194 | 0 | 0 | 0 | -3.18739 | 0.984805 | -15.2813 | 4.79818 | 0.02849 |
| 249 | 2.67226 | 0 | 0 | 0 | -3.11776 | 0.959283 | -14.5343 | 5.93046 | 0.014881 |
| 251 | 2.41115 | 0 | 0 | 0 | -2.81312 | 0.825653 | -13.6096 | 5.23172 | 0.022179 |
| 252 | 5.7123 | 0 | 0 | 0 | -6.66461 | 2.20839 | -19.1388 | 10.9087 | 0.000957 |
| 253 | 3.5043 | 0 | 0 | 0 | -4.08851 | 1.22017 | -15.6894 | 7.68208 | 0.005577 |
| 254 | 4.53262 | 0 | 0 | 0 | -5.28826 | 1.59054 | -18.912 | 8.25339 | 0.004068 |
| 255 | 2.83915 | 0 | 0 | 0 | -3.31247 | 0.811718 | -15.8364 | 6.13541 | 0.01325 |
| 260 | 2.63499 | 0 | 0 | 0 | -3.07428 | 1.22961 | -15.257 | 6.0081 | 0.01424 |
| 262 | 6.61098 | 0 | 0 | 0 | -7.71312 | 1.55468 | -18.8804 | 10.5661 | 0.001152 |
| 263 | 3.26794 | 0 | 0 | 0 | -3.81275 | 1.12582 | -13.7399 | 5.98004 | 0.014469 |
| 265 | 3.85814 | 0 | 0 | 0 | -4.50135 | 1.79151 | -12.1859 | 6.31698 | 0.011959 |
| 266 | 4.9894 | 0 | 0 | 0 | -5.82119 | 1.29653 | -11.1075 | 5.71841 | 0.016788 |
| 267 | 2.69183 | 0 | 0 | 0 | -3.14059 | 0.930538 | -12.796 | 5.03827 | 0.024793 |
| 269 | 4.65968 | 0 | 0 | 0 | -5.43651 | 2.30997 | -21.212 | 7.92552 | 0.004874 |
| 270 | 4.2376 | 0 | 0 | 0 | -4.94407 | 1.34546 | -10.8387 | 4.39676 | 0.036007 |
| 271 | 3.03661 | 0 | 0 | 0 | -3.54285 | 0.890916 | -15.0103 | 5.6938 | 0.017025 |
| 273 | 2.93852 | 0 | 0 | 0 | -3.42842 | 1.14761 | -14.7119 | 5.91154 | 0.015042 |
| 274 | 3.42648 | 0 | 0 | 0 | -3.99772 | 1.88727 | -20.003 | 6.2549 | 0.012385 |
| 284 | 1.86678 | 0 | 0 | 0 | -2.178 | 0.931271 | -14.515 | 5.14118 | 0.023365 |
| 292 | 2.06189 | 0 | 0 | 0 | -2.40564 | 0.87563 | -11.7277 | 4.51403 | 0.033618 |
| 295 | 2.85455 | 0 | 0 | 0 | -3.33044 | 1.16907 | -15.6782 | 6.98837 | 0.008204 |
| 298 | 6.01578 | 0 | 0 | 0 | -7.01869 | 1.49505 | -11.9677 | 6.53836 | 0.010557 |
| 299 | 1.79496 | 0 | 0 | 0 | -2.09421 | 0.814989 | -16.2157 | 4.06695 | 0.04373 |
| 309 | 7.7692 | 0 | 0 | 0 | -9.06443 | 1.5959 | -19.3644 | 8.35269 | 0.003851 |
| 311 | 4.11673 | 0 | 0 | 0 | -4.80304 | 1.26464 | -10.1271 | 5.2582 | 0.021844 |
| 317 | 4.60992 | 0 | 0 | 0 | -5.37846 | 2.27223 | -22.3982 | 7.87242 | 0.005019 |
| 319 | 4.93989 | 0 | 0 | 0 | -5.76343 | 1.84477 | -12.1573 | 7.16894 | 0.007418 |
| 323 | 3.53742 | 0 | 0 | 0 | -4.12716 | 1.39933 | -15.7657 | 7.22227 | 0.0072 |
| 330 | 2.25343 | 0 | 0 | 0 | -2.62911 | 0.887168 | -14.2278 | 5.47754 | 0.019262 |
| 340 | 7.26147 | 0 | 0.496887 | 0 | -8.47206 | 1.63362 | -19.905 | 7.42543 | 0.006431 |
| 341 | 3.76489 | 0 | 0 | 0 | -4.39255 | 0.812701 | -10.3109 | 3.94794 | 0.046929 |
| 348 | 2.53284 | 0 | 0 | 0 | -2.9551 | 1.02767 | -13.7595 | 6.5254 | 0.010635 |
| 350 | 3.12884 | 0 | 0.315571 | 0 | -3.65046 | 0.891162 | -19.6825 | 5.67051 | 0.017252 |
| 352 | 1.52526 | 0 | 0 | 0 | -1.77954 | 0.876725 | -13.0124 | 4.03413 | 0.044589 |
| 358 | 5.3755 | 0 | 0 | 0 | -6.27167 | 1.54269 | -10.4054 | 6.63404 | 0.010005 |
| 359 | 3.21552 | 0 | 0.361634 | 0 | -3.75159 | 1.06728 | -20.4529 | 5.93013 | 0.014884 |
| 363 | 7.16114 | 0 | 0 | 0 | -8.355 | 1.21581 | -18.6332 | 9.2439 | 0.002363 |
| 367 | 2.50468 | 0 | 0 | 0 | -2.92225 | 1.32174 | -18.6615 | 6.29035 | 0.01214 |
| 371 | 6.01537 | 0 | 0 | 0 | -7.01821 | 2.14953 | -19.0597 | 9.97666 | 0.001585 |
| 372 | 8.0589 | 0 | 0 | 0 | -9.40243 | 2.20146 | -16.7846 | 11.0065 | 0.000908 |
| 373 | 2.4633 | 0 | 0 | 0 | -2.87396 | 1.41146 | -18.3387 | 5.01197 | 0.025173 |
| 378 | 2.63586 | 0 | 0 | 0 | -3.0753 | 1.04598 | -15.7717 | 6.25505 | 0.012384 |
| 392 | 2.4304 | 0 | 0 | 0 | -2.83558 | 0.804555 | -12.302 | 5.18585 | 0.022772 |
| 393 | 4.08339 | 0 | 0 | 0 | -4.76414 | 0.727332 | -11.2416 | 4.61463 | 0.0317 |
| 394 | 6.14237 | 0 | 0 | 0 | -7.16639 | 1.69403 | -15.1982 | 9.4572 | 0.002103 |
| 396 | 3.61535 | 0 | 0 | 0 | -4.21807 | 2.03763 | -24.7056 | 6.53096 | 0.010601 |
| 397 | 1.69733 | 0 | 0 | 0 | -1.9803 | 0.615091 | -12.4686 | 3.98334 | 0.045952 |
| 399 | 3.74756 | 0 | 0 | 0 | -4.37233 | 1.35197 | -11.973 | 5.64238 | 0.017531 |
| 405 | 4.91448 | 0 | 0 | 0 | -5.73379 | 1.96054 | -27.7642 | 9.64313 | 0.001901 |
| 408 | 4.15037 | 0 | 0 | 0 | -4.84229 | 1.70098 | -12.7237 | 4.99898 | 0.025362 |
| 409 | 3.52972 | 0 | 0 | 0 | -4.11817 | 1.25509 | -18.5167 | 7.93673 | 0.004844 |
| 416 | 3.6295 | 0 | 0 | 0 | -4.23458 | 1.49327 | -21.4744 | 6.49129 | 0.01084 |

| | | | | | | | | | |
|---|---|---|---|---|---|---|---|---|---|
| 419 | 16.4588 | 0 | 0 | 0 | -19.2027 | 1.637 | -25.3227 | 15.1846 | 9.75E-05 |
| 423 | 3.45181 | 0 | 0 | 0 | -4.02727 | 1.11469 | -15.4653 | 7.20988 | 0.00725 |
| 424 | 4.46152 | 0 | 0 | 0 | -5.20531 | 1.18539 | -17.6491 | 7.76891 | 0.005315 |
| 432 | 4.0907 | 0 | 0 | 0 | -4.77267 | 1.9813 | -20.9717 | 7.00961 | 0.008107 |
| 437 | 2.56825 | 0 | 0 | 0 | -2.99641 | 0.948239 | -13.8763 | 5.80833 | 0.01595 |
| 438 | 11.6484 | 0 | 0 | 0 | -13.5904 | 2.53209 | -24.1889 | 13.1119 | 0.000293 |
| 439 | 7.32208 | 0 | 0 | 0 | -8.54277 | 2.8696 | -23.9462 | 9.37149 | 0.002204 |
| 441 | 2.75639 | 0 | 0 | 0 | -3.21591 | 1.00664 | -14.1662 | 7.07256 | 0.007827 |
| 442 | 2.43141 | 0 | 0 | 0 | -2.83676 | 0.92639 | -14.4408 | 5.60865 | 0.017872 |
| 446 | 14.1636 | 0.767609 | 0 | 0.054 | -15.6293 | 3.67913 | -18.916 | 8.1329 | 0.004347 |
| 458 | 5.11642 | 0 | 0 | 0 | -5.9694 | 1.71445 | -18.1394 | 10.3823 | 0.001272 |
| 465 | 3.96484 | 0 | 0 | 0 | -4.62583 | 1.70797 | -13.6646 | 6.27439 | 0.01225 |
| 469 | 2.73052 | 0 | 0 | 0 | -3.18573 | 1.04779 | -13.9871 | 6.76036 | 0.00932 |
| 471 | 3.90025 | 0 | 0 | 0 | -4.55047 | 0.921989 | -10.8707 | 6.4906 | 0.010845 |
| 474 | 2.12742 | 0 | 0.518036 | 0 | -2.48208 | 1.24266 | -21.3758 | 4.0894 | 0.043153 |
| 479 | 4.28427 | 0 | 0.864453 | 0 | -4.99851 | 1.73259 | -28.9757 | 8.29179 | 0.003982 |
| 480 | 5.66189 | 0 | 0 | 0 | -6.60581 | 0.917161 | -15.4668 | 6.44745 | 0.011111 |
| 483 | 5.66562 | 0 | 0 | 0 | -6.61015 | 1.49161 | -11.2052 | 6.15701 | 0.013089 |
| 487 | 2.86717 | 0 | 0 | 0 | -3.34517 | 0.774695 | -15.7144 | 5.86704 | 0.015427 |
| 496 | 2.92905 | 0 | 0 | 0 | -3.41737 | 1.28306 | -19.9601 | 6.4001 | 0.011411 |
| 500 | 2.90431 | 0 | 0 | 0 | -3.3885 | 0.679256 | -10.1297 | 3.84751 | 0.04982 |
| 505 | 2.72973 | 0 | 0 | 0 | -3.18481 | 0.587906 | -11.2014 | 4.20364 | 0.040337 |
| 529 | 4.13961 | 0 | 0 | 0 | -4.82973 | 1.1489 | -10.6521 | 5.24949 | 0.021953 |
| 531 | 6.98014 | 0 | 0 | 0 | -8.14382 | 1.21E-16 | -17.1888 | 7.60396 | 0.005824 |
| 533 | 12.9124 | 0 | 0.652744 | 0 | -15.0651 | 1.87491 | -17.8773 | 6.38464 | 0.011511 |
| 547 | 2.70397 | 0 | 0.497279 | 0 | -3.15476 | 1.68443 | -32.6017 | 5.40066 | 0.020129 |
| 550 | 9.14258 | 0 | 0 | 0 | -10.6668 | 1.34291 | -13.6707 | 9.7576 | 0.001786 |
| 564 | 7.65588 | 0 | 0 | 0 | -8.93222 | 3.51266 | -24.4179 | 11.7754 | 0.0006 |
| 565 | 6.96159 | 0 | 0 | 0 | -8.12218 | 1.60932 | -11.3823 | 7.72821 | 0.005436 |
| 566 | 17.015 | 0 | 0 | 0 | -19.8516 | 3.46801 | -13.2832 | 12.0227 | 0.000526 |
| 568 | 22.6775 | 0 | 0 | 0 | -26.4581 | 1.30309 | -18.8398 | 13.3975 | 0.000252 |
| 571 | 2.03008 | 0 | 0 | 0 | -2.36853 | 1.14057 | -15.8894 | 5.87312 | 0.015374 |
| 574 | 22.0727 | 0 | 0.350623 | 0 | -25.7525 | 2.32731 | -20.9033 | 14.1677 | 0.000167 |
| 577 | 10.0944 | 0 | 0.475827 | 0 | -11.7773 | 1.68954 | -21.9156 | 8.73107 | 0.003128 |
| 578 | 1.8942 | 0 | 0 | 0 | -2.20999 | 1.23929 | -20.3047 | 3.90851 | 0.048042 |
| 579 | 4.45519 | 0 | 0 | 0 | -5.19793 | 1.74175 | -13.2622 | 5.17459 | 0.02292 |

**Table S9** Negative selection sites for the N gene based on FEL analysis.

| Codon | dS | dN | dN Leaves | dN/dS | Normalize dN-dS | dS (when dN=dS) | Log(L) | LRT | P value |
|---|---|---|---|---|---|---|---|---|---|
| 12 | 4.17546 | 0 | 0 | 0 | -6.79425 | 1.82333 | -15.1062 | 4.62258 | 0.031554 |
| 27 | 9.23733 | 0 | 0.596667 | 0 | -15.0309 | 1.88208 | -15.5064 | 5.27656 | 0.021614 |
| 40 | 15.1261 | 0 | 0 | 0 | -24.6131 | 1.20623 | -8.82357 | 4.06481 | 0.043786 |
| 51 | 8.34415 | 0 | 0 | 0 | -13.5775 | 2.70208 | -12.0468 | 5.343 | 0.020806 |
| 53 | 3.77345 | 0 | 0 | 0 | -6.14011 | 2.25096 | -14.5155 | 5.01297 | 0.025158 |
| 59 | 3.50725 | 0 | 0 | 0 | -5.70696 | 1.98818 | -14.2683 | 4.26081 | 0.039001 |
| 63 | 31.9659 | 1.19105 | 0 | 0.037 | -50.0765 | 4.0125 | -14.0284 | 5.86068 | 0.015483 |
| 67 | 5.00607 | 0 | 0 | 0 | -8.14582 | 1.92171 | -13.1325 | 4.87395 | 0.027265 |
| 78 | 9.02098 | 0 | 1.09915 | 0 | -14.6788 | 3.1453 | -20.7008 | 6.15029 | 0.013139 |
| 83 | 4.94794 | 0 | 0 | 0 | -8.05123 | 1.56487 | -13.8078 | 5.97564 | 0.014505 |
| 86 | 6.09347 | 0 | 0.715115 | 0 | -9.91523 | 2.50798 | -18.6943 | 5.74064 | 0.016577 |
| 88 | 15.2044 | 0 | 0 | 0 | -24.7405 | 1.77652 | -9.91385 | 6.06861 | 0.013761 |
| 95 | 6.89381 | 0 | 0.595592 | 0 | -11.2175 | 3.27499 | -20.5155 | 6.25804 | 0.012363 |
| 97 | 26.4993 | 0 | 0 | 0 | -43.1194 | 3.58116 | -17.3611 | 9.77725 | 0.001767 |
| 100 | 5.27271 | 0 | 0.614496 | 0 | -8.57969 | 2.08862 | -17.3401 | 4.93021 | 0.026391 |
| 102 | 3.9478 | 0 | 0 | 0 | -6.42381 | 1.73007 | -13.6829 | 4.65231 | 0.031012 |
| 105 | 5.93287 | 0 | 0 | 0 | -9.6539 | 1.89893 | -16.9065 | 5.86511 | 0.015444 |
| 125 | 6.22325 | 0 | 0.422375 | 0 | -10.1264 | 2.37976 | -22.7647 | 5.33564 | 0.020894 |
| 127 | 5.0986 | 0 | 0 | 0 | -8.29639 | 1.08173 | -10.2751 | 3.99354 | 0.045675 |
| 142 | 5.22845 | 0 | 0.383734 | 0 | -8.50767 | 2.41015 | -23.3554 | 5.34289 | 0.020807 |
| 187 | 2.12989 | 0 | 0 | 0 | -3.46574 | 1.01754 | -15.0495 | 4.13977 | 0.041887 |
| 190 | 2.29851 | 0 | 0 | 0 | -3.7401 | 0.87253 | -12.1307 | 4.53171 | 0.033272 |
| 192 | 14.374 | 0 | 0 | 0 | -23.3892 | 1.67381 | -14.8026 | 11.5439 | 0.00068 |
| 198 | 2.10075 | 0 | 0 | 0 | -3.41832 | 1.07663 | -14.9748 | 4.93342 | 0.026342 |

| | | | | | | | | | |
|---|---|---|---|---|---|---|---|---|---|
| 206 | 4.38981 | 0 | 0.452736 | 0 | -7.14305 | 1.0201 | -15.1539 | 5.04573 | 0.024687 |

**Table S10** Negative selection sites for the 7b gene based on FEL analysis.

| Codon | dS | dN | dN Leaves | dN/dS | Normalized dN-dS | dS (when dN=dS) | Log(L) | LRT | p value |
|---|---|---|---|---|---|---|---|---|---|
| 18 | 4.32222 | 0.54684 | 0.568281 | 0.127 | -5.34823 | 1.87589 | -21.8218 | 4.22918 | 0.039735 |
| 31 | 8.61168 | 0 | 0 | 0 | -12.1994 | 1.1671 | -16.1834 | 9.53625 | 0.002015 |
| 35 | 3.7082 | 0 | 0 | 0 | -5.25307 | 2.25648 | -20.8911 | 4.24847 | 0.039286 |
| 38 | 2.63499 | 0 | 0 | 0 | -3.73275 | 0.80128 | -10.598 | 4.01758 | 0.045028 |
| 48 | 15.1646 | 0 | 0 | 0 | -21.4823 | 1.59602 | -10.726 | 8.69746 | 0.003187 |
| 51 | 3.90024 | 0 | 0 | 0 | -5.52511 | 2.28983 | -18.6977 | 4.49845 | 0.033926 |
| 56 | 5.95925 | 0 | 0 | 0 | -8.44192 | 2.18816 | -12.483 | 3.88362 | 0.048759 |
| 57 | 24.6266 | 0 | 1.29625 | 0 | -34.8862 | 3.19798 | -22.3248 | 11.8525 | 0.000576 |
| 63 | 12.3311 | 0 | 0 | 0 | -17.4684 | 2.2831 | -9.25505 | 6.24571 | 0.012449 |
| 72 | 4.0724 | 0 | 0 | 0 | -5.76899 | 1.25042 | -10.8357 | 4.18348 | 0.04082 |
| 81 | 7.81155 | 0 | 0 | 0 | -11.0659 | 3.48565 | -11.0632 | 6.89682 | 0.008635 |
| 90 | 11.001 | 0 | 0 | 0 | -15.5841 | 2.05727 | -8.65598 | 4.42292 | 0.035459 |
| 109 | 9.14246 | 0 | 0 | 0 | -12.9513 | 2.91767 | -10.923 | 5.47904 | 0.019246 |
| 111 | 5.317 | 0 | 0 | 0 | -7.5321 | 2.30418 | -12.7114 | 4.41152 | 0.035697 |
| 132 | 8.30444 | 0 | 0 | 0 | -11.7641 | 1.76574 | -11.1725 | 5.53135 | 0.018679 |
| 141 | 9.24906 | 0 | 0 | 0 | -13.1023 | 2.2522 | -12.6529 | 5.4615 | 0.01944 |
| 168 | 5.51604 | 0 | 0 | 0 | -7.81407 | 1.24005 | -9.58977 | 4.17216 | 0.041094 |
